# Properties for Thermally Conductive Interfaces with Wide Band Gap Materials

**DOI:** 10.1021/acsami.2c01351

**Published:** 2022-07-27

**Authors:** Samreen Khan, Frank Angeles, John Wright, Saurabh Vishwakarma, Victor H. Ortiz, Erick Guzman, Fariborz Kargar, Alexander A. Balandin, David J. Smith, Debdeep Jena, H. Grace Xing, Richard Wilson

**Affiliations:** † University of California Riverside, Riverside, California 92521, United States; ‡ Cornell University, Ithaca, New York 14850, United States; § Arizona State University, Tempe, Arizona 85287, United States

**Keywords:** Ultra-wide band gap semiconductors, thermal interface conductance, thermal boundary resistance, phonons, time-domain thermoreflectance

## Abstract

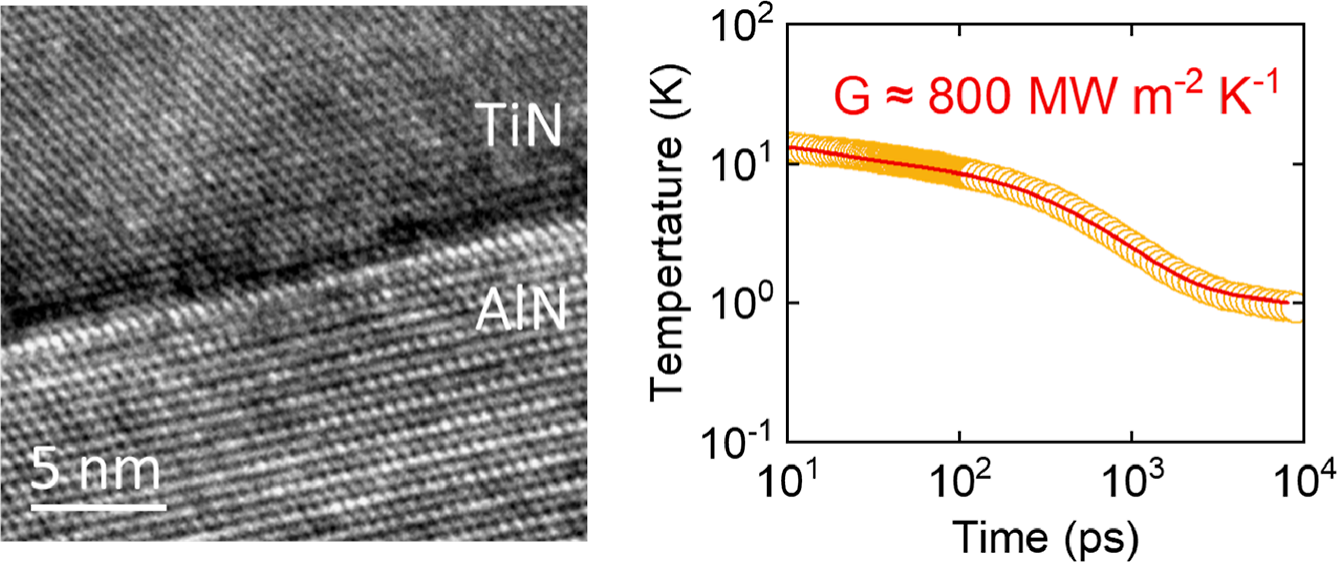

The goal of this study is to determine how bulk vibrational properties and interfacial structure affect thermal transport at interfaces in wide band gap semiconductor systems. Time-domain thermoreflectance measurements of thermal conductance *G* are reported for interfaces between nitride metals and group IV (diamond, SiC, Si, and Ge) and group III–V (AlN, GaN, and cubic BN) materials. Group IV and group III–V semiconductors have systematic differences in vibrational properties. Similarly, HfN and TiN are also vibrationally distinct from each other. Therefore, comparing *G* of interfaces formed from these materials provides a systematic test of how vibrational similarity between two materials affects interfacial transport. For HfN interfaces, we observe conductances between 140 and 300 MW m^–2^ K^–1^, whereas conductances between 200 and 800 MW m^–2^ K^–1^ are observed for TiN interfaces. TiN forms exceptionally conductive interfaces with GaN, AlN, and diamond, that is, *G* > 400 MW m^–2^ K^–1^. Surprisingly, interfaces formed between vibrationally similar and dissimilar materials are similarly conductive. Thus, vibrational similarity between two materials is not a necessary requirement for high *G*. Instead, the time-domain thermoreflectance experiment (TDTR) data, an analysis of bulk vibrational properties, and transmission electron microscopy (TEM) suggest that *G* depends on two other material properties, namely, the bulk phonon properties of the vibrationally softer of the two materials and the interfacial structure. To determine how *G* depends on interfacial structure, TDTR and TEM measurements were conducted on a series of TiN/AlN samples prepared in different ways. Interfacial disorder at a TiN/AlN interface adds a thermal resistance equivalent to ∼1 nm of amorphous material. Our findings improve fundamental understanding of what material properties are most important for thermally conductive interfaces. They also provide benchmarks for the thermal conductance of interfaces with wide band gap semiconductors.

## Introduction

Devices made from wide band gap semiconductors can outperform their silicon-based counterparts.^1^ For example, high voltage SiC devices have higher breakdown voltages and higher on/off ratios than comparable Si devices.^2^ Additionally, SiC has a thermal conductivity 3 times larger than that of Si,^3,4^ which aids thermal performance. Due to better thermal performance, SiC devices have aided in the development of green technologies such as electric vehicles^5^ and wind turbines.^6^ Wide band gap materials also offer advantages for high-frequency telecommunications electronics.^7,8^ The high critical field of GaN allows high-frequency GaN electronics to outperform RF devices consisting of small band gap materials.^7^ Further gains in performance of high-power and high-frequency electronics require semiconductor materials with even wider band gaps than SiC or GaN.^8^ Therefore, there is an urgent need to understand the materials physics of ultra-wide band gap materials such as AlN, Ga_2_O_3_, cubic BN, and diamond.^8^ The focus of this study is to advance fundamental understanding of interfacial heat transfer in wide and ultra-wide band gap material systems.

Understanding the physics of interfacial thermal transport is critical because thermally resistive interfaces limit the maximum power before device failure.^9^ Thermally resistive interfaces also hinder device reliability.^10^ The effect of interfaces on thermal transport is characterized by the interfacial thermal conductance per unit area *G*. The conductance *G* relates the heat current at the interface, *J*, to the temperature drop, Δ*T*, at the interface, *J* = −*G*Δ*T*. Decades of research have firmly established the phenomenology of *G* in small band gap material systems, for example, Si.^11^ However, an experimental understanding of *G* in wide and ultra-wide band gap materials is emerging only now.^12−14^

Recent studies have shown that strong chemical bonds are a pre-requisite for conductive interfaces in GaN,^15^ Ga_2_O_3_,^14^ SiC,^15^ and diamond systems.^16,17^ Research has also shown that interfacial chemical reactions can lead to larger *G*,^17,18^ while a few nanometers of crystalline disorder at the interface leads to lower *G*.^15^ Despite these advances, important gaps in the fundamental understanding of *G* remain. These include the effect on *G* of: (i) vibrational similarity of the materials forming the interface; (ii) complex unit cells; and (iii) interfacial structure. A fundamental understanding of vibrational similarity and complex unit cells is particularly important for high-power electronic applications. Candidate device heterostructures for high power applications often involve heterostructures composed of vibrationally dissimilar materials, for example, AlGaN/GaN/diamond.^19^ Other device heterostructures involve materials with complex unit cells, such as β-Ga_2_O_3_ or 4H-SiC.^8^

**Figure 1:**
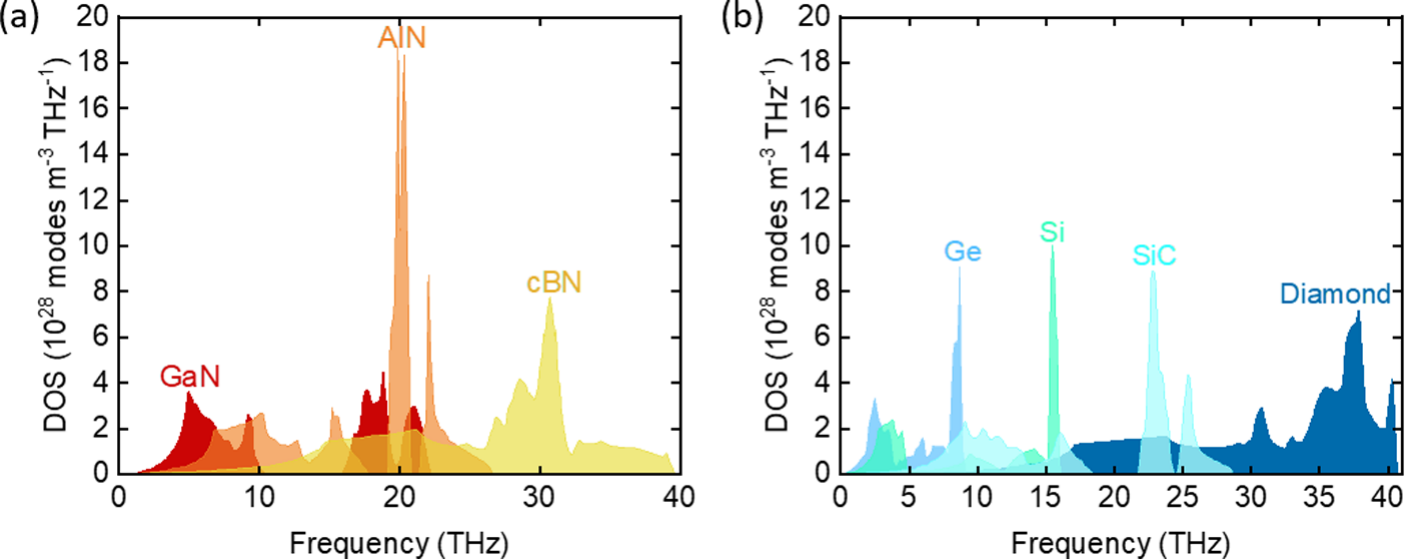
Density of states for (a) GaN, AlN, and cBN and (b) diamond, SiC-3C, Si, and Ge. As average atomic mass decreases, the vibrational spectrum stiffens, that is, frequencies increase.

Nitride metals are among the most vibrationally stiff metals. Their vibrational stiffness allows one to study the upper limits to the interface conductance of wide and ultra-wide band gap materials. Here, we report time-domain thermoreflectance (TDTR) measurements of the interface conductance between nitride metals and group IV materials: diamond, SiC, Si, and Ge. We also measure transport between nitride metals and group III–V crystals: AlN, GaN, and cubic BN. The group IV and group III–V materials have systematic differences in acoustic properties; see Figure 1. These differences allow tests of how vibrational similarity between the nitride metal and a specific substrate affects interfacial transport. We compare isovalent materials (cBN to AlN to GaN and diamond to SiC to Si to Ge) in an effort to minimize variations in *G* that occur because of changes in interfacial bonding strength or interfacial chemical reactions.

## Experimental Section

### Sample Preparation

Nitride metal films were deposited using reactive DC magnetron sputtering in a mixed N_2_/Ar environment using an AJA Orion Series sputtering system. Because oxygen has a detrimental effect on the transport properties of nitride metals, we took several steps to reduce oxygen content in the chamber prior to deposition. To reduce the pressure of the sputtering chamber below 3 × 10^–7^ Torr, it was baked at approximately 100 °C for at least 12 h prior to deposition. Prior to deposition, titanium was sputtered as an oxygen getter material for 10 min. The base pressure of the chamber was further reduced by circulating liquid nitrogen through coils within the chamber.

Prior to deposition of the nitride metal, the substrates were subjected to heat and plasma treatments. Substrates were heated to ≈450 °C for 20 min in order to vaporize hydrocarbons or other physisorbed molecules present on the surface. The heating temperature of the substrates is only approximate because the temperature was measured at the heating source rather than the sample stage. Then, the substrates were RF sputter-etched at 3.5 mTorr and 35 W for 5 min in an effort to increase the strength of interfacial bonds between the nitride metal and substrate. Brief RF sputter etching has been shown previously to change interfacial bonding and enhance interfacial thermal transport at diamond surfaces.^20^

The deposition temperatures and N_2_/Ar partial pressures were selected through an iterative process that used refs (21) and (22) as guidelines. The deposition parameters listed in Table 1 reliably resulted in polycrystalline TiN and HfN films with good electrical conductivity. In Supporting Information, atomic force microscopy (AFM) scans of the TiN surface morphology are shown, as well as Raman spectra from the TiN and HfN films.

**Table 1:** Deposition Parameters for TiN and HfN Films

nitride metal	deposition temp (°C)	sputtering pressure (mTorr)	nitrogen partial pressure (mTorr)	argon partial pressure (mTorr)
hafnium nitride	550	3.5	0.2	3.3
titanium nitride	575	1.3	0.6	0.7

The TiN and HfN films were deposited on group IV crystals (diamond, SiC, Si, and Ge), group III–V crystals (AlN, GaN, and cubic BN), and oxide crystals (Al_2_O_3_ and MgO). TiN and HfN were also deposited on Si with (100) orientation and SiO_2_/Si with (100) orientation wafers from University Wafers as control samples. The (0001) 4H-SiC substrate, (0001) 6H-SiC substrate, (100) Ge substrate, (0001) GaN film on sapphire, and (100) 3C-SiC film on Si were purchased from MTI Corporation. Cubic BN crystals were purchased from Hyperion Materials & Technologies, Inc.

In addition to sputter-deposited TiN/AlN samples prepared as described above, we studied a TiN/AlN interface prepared via MBE. The growth of TiN on AlN films was performed in a Veeco GENxplor system with a base pressure less than 1 × 10^–10^ Torr. The entire heterostructure consisted of a *c*-plane sapphire wafer, 45 nm Nb_2_N, 660 nm AlN, and 68 nm TiN films. The films were grown by plasma-assisted molecular beam epitaxy (PAMBE) on a 1 cm^2^*c*-plane sapphire substrate. Nb and Ti were supplied using an e-beam evaporator, with flux measured and controlled using an electron impact emission spectroscopy (EIES) system. The Nb_2_N film was grown at a substrate temperature of 1150 °C, with an active nitrogen flux that exceeded the Nb flux. Growth at these temperatures is found to yield single crystal β-Nb_2_N films, with the *c*-axis aligned to the sapphire *c*-axis. The AlN film was grown by nucleating the film growth with an active nitrogen flux, which exceeds the aluminum flux, which was discovered to be necessary to prevent reaction of any excess aluminum with the underlying Nb_2_N. This nucleation was approximately 100 nm in thickness and is grown at 725 °C. The remaining AlN growth is completed at 825 °C with an aluminum flux greater than the active nitrogen flux, such that aluminum droplets accumulate on the sample surface, which is a well-known growth condition to yield high crystal quality and low roughness AlN grown by MBE.^23^ Prior to growth of the TiN layer, all accumulated aluminum droplets are thermally desorbed by heating the substrate to 1000 °C, a temperature that gives rapid evaporation of liquid Al. The TiN was then grown at a substrate temperature of 1000 °C. The TiN was grown under nitrogen-rich conditions at a growth rate of approximately 2.3 nm/min.

### Material Characterization

To characterize samples after synthesis, we did transmission electron microscopy (TEM), atomic force microscopy, Raman spectroscopy, and electrical resistivity measurements. Atomic force microscopy revealed surface roughness of diamond, CVD AlN, polycrystal AlN, Al_2_O_3_, MgO, and GaN substrates to be between 0.2 and 2 nm. Raman spectra were performed on HfN and TiN on Si and MgO substrates in the backscattering configuration using 633 nm (red) laser excitation wavelength with a cutoff frequency of 110 cm^–1^. The Raman spectra are consistent with stoichiometric growth of TiN on both Si and MgO substrates (see Supporting Information). The electrical resistivity of all nitride films was measured with the four-point probe method. Typical resistivity values for the TiN and HfN films were 30–70 μΩcm but varied with substrate (see Supporting Information). TEM provided measurements of film thickness and interfacial structure. Samples suitable for observation in cross section by TEM were prepared using a dual-beam Helios G5UX system. Observations were made using a Phillips-FEI CM200 FEG-TEM operated at 200 kV and an image-corrected FEI Titan 80-300 operated at 300 kV.

### Time-Domain Thermoreflectance

TDTR is an optical pump/probe technique used to measure thermal transport properties in thin films and at interfaces.^24^ The pump beam heats up the metal transducer, for example, TiN or HfN metal, resulting in transient evolution of the surface temperature. A time-delayed probe pulse measures the fluctuation of the sample’s reflectance caused by the change in surface temperature. The wavelength of the laser in these experiments is centered at 783 nm, and the pump beam is modulated at 10.7 MHz. The intensity of the reflected probe beam is measured using a silicon photodetector. Optical filters are used to prevent the reflected pump beam from reaching the detector. The photodiode is connected to an RF lock-in amplifier, which measures in-phase and out-of-phase voltages as a function of delay time. The ratio of the in- and out-of-phase temperature response to pump heating is then determined. Further details on the experimental setup can be found elsewhere.^25^

**Figure 2:**
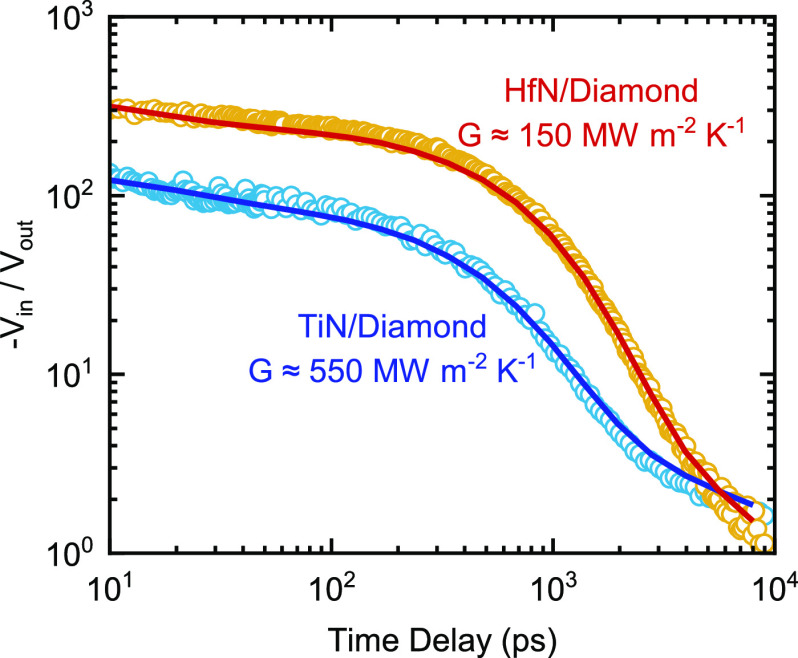
Time-domain thermoreflectance data for 54 nm TiN/diamond and 35 nm HfN/diamond samples. Lines are predictions of a thermal model with an interface conductance of 550 MW m^–2^ K^–1^ for TiN/diamond and 150 MW m^–2^ K^–1^ for HfN/diamond. The blue curve for TiN/diamond decays at a higher rate than the red curve for HfN/diamond due to higher interface conductance.

Experimental TDTR signals are compared to the predictions of a thermal model, which is a 3D analytical solution to the heat diffusion equation for a multilayer structure.^24^ The thermal model uses the thermal conductivity, heat capacity, and the thickness of each layer as inputs and predicts the corresponding temperature response. Unknown thermal properties are adjusted until the predictions of the thermal model agree with the experimental data. Literature values for the heat capacities of TiN, HfN, and all the substrates were used as inputs for the model. The metal thicknesses and thermal conductivity were fixed as described in Supporting Information. The interfacial thermal conductance and substrate thermal conductivity were treated as fit parameters. Figure 2 shows data from the TDTR measurements for 54 nm of TiN and 35 nm of HfN deposited on diamond substrates. Figure 2 also shows the corresponding fit with the thermal model. The measured conductances of the TiN/diamond interface and the HfN/diamond interface are 550 and 150 MW/m^2^ K, respectively.

## Results and Discussion

### Effect of Substrate Vibrational Properties on *G*

**Figure 3:**
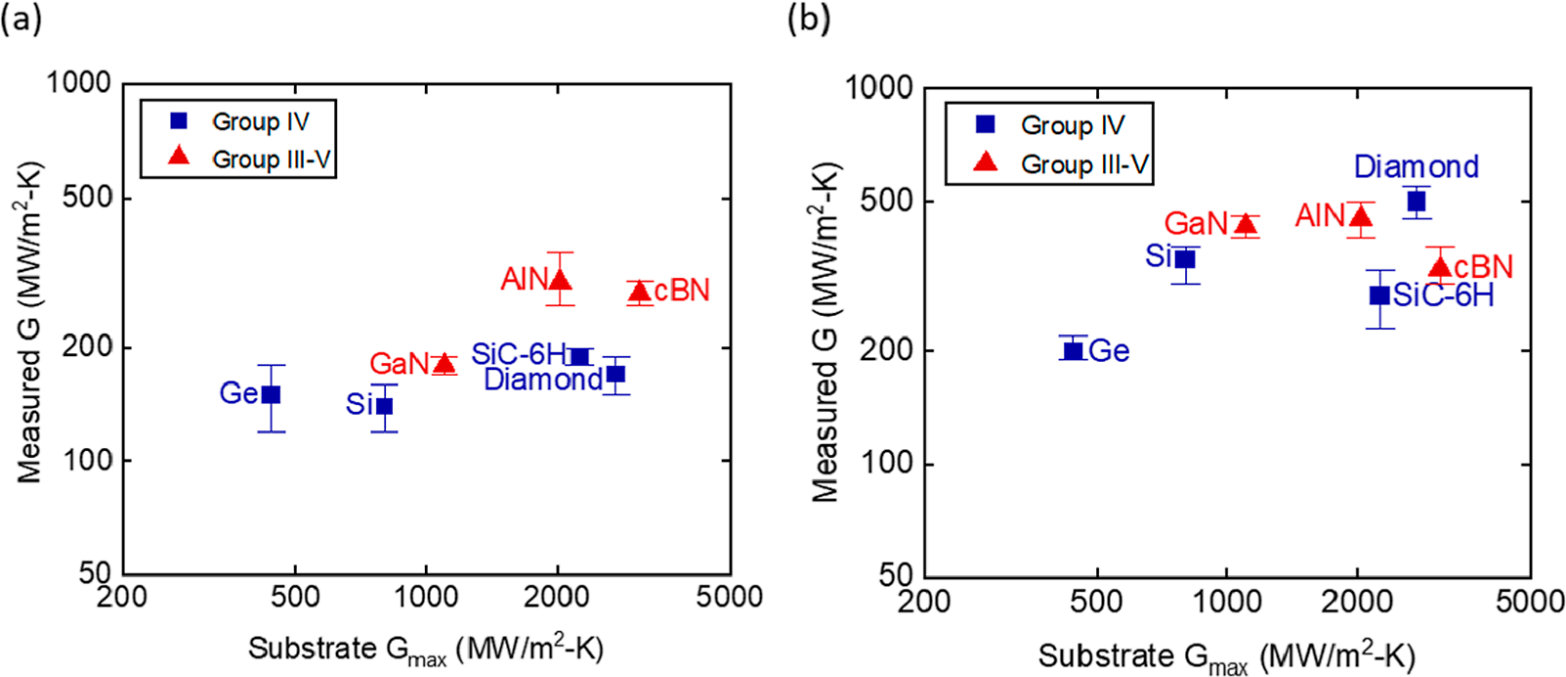
Interface conductance between (a) HfN and (b) TiN and group IV materials and group III–V materials as a function of *G*_max_ of the substrates. The error bars reflect high and low conductance values based on the uncertainty in thickness of the nitride metal layer.

Irrespective of the vibrational properties of the substrate, all studied HfN interfaces have comparable heat-carrying abilities. The interface conductance of HfN and the group IV semiconductors ranges between 140 and 200 MW m^–2^ K^–1^; see Figure 3a. The interface conductance of HfN and the group III–V semiconductors range from 170 to 300 MW m^–2^ K^–1^. To quantify differences in the vibrational properties in the semiconductors studied, *G*_max_ was calculated for each group IV and III–V material. *G*_max_ of a material (equivalent to the maximum transmission model or limit in refs (26) and (27)) is the kinetic theory prediction for the thermal conductance of an interface with that material.^26,28^*G*_max_ is correlated with the heat-carrying abilities of the material’s phonons. TDTR measurements show that HfN forms equally conductive interfaces with Ge and diamond (Figure 3a) despite diamond being six times as vibrationally stiff as Ge (*G*_max_ ≈ 3000 vs 500 MW m^–2^ K^–1^).

In contrast to HfN, TiN forms highly conductive interfaces with the wide and ultra-wide band gap semiconductors. For example, Figure 3b shows experimentally measured values of *G* between TiN and group IV materials and group III–V materials plotted as a function of *G*_max_ of the substrate. For the TiN/group IV semiconductors, a weak positive correlation is observed between *G* and *G*_max_. An increase in *G* from ≈200 ± 20 MW m^–2^ K^–1^ for TiN/Ge to ≈500 ± 50 MW m^–2^ K^–1^ for TiN/diamond is observed. Alternatively, for group III–IV materials, there is no significant correlation between *G* and the vibrational properties of the substrate, that is, *G* and *G*_max_. *G* for TiN/group III–V nitrides ranges from 300 to 500 MW m^–2^ K^–1^.

To evaluate and confirm the reproducibility of the interface conductance values for several material systems, as reported in Figures 2 and 3, multiple samples were prepared. For example, three samples were measured for TiN/diamond system. In these samples, the highest *G* of ≈550 ± 100 MW m^–2^ K^–1^ was observed between TiN and a high-purity element-six ELSC grade {100} diamond. A similar value was measured for *G* of ≈500 ± 40/90 MW m^–2^ K^–1^ between TiN and a lower-grade element-six {100} CVD diamond substrate. Finally, a third TiN/diamond sample was prepared after wet-etch removal of the original TiN layer on the lower-grade element-six diamond. To remove the TiN film, the sample was dipped in HF for ∼10 s; the sample was then dipped in a mixture of 4:1 KOH/H_2_O_2_ at 70 °C, for approximately 10 min until the TiN had been etched. Diamond is notoriously resistant to wet chemical etching.^29^ Nevertheless, the TiN on wet-etched diamond had the lowest *G* of ≈320 ± 30 MW m^–2^ K^–1^. The extra thermal resistance at the TiN/wet-etched diamond interface was equivalent to resistance from ∼1 nm of amorphous carbon with a thermal conductivity of 1 W m^–1^ K^–1^.

In addition to the group III–V and group IV substrates, conductance between the nitride metals and sapphire and MgO was also measured. The *G* for TiN/MgO ≈ 550 ± 50 MW m^–2^ K^–1^, HfN/MgO ≈ 200 ± 20 MW m^–2^ K^–1^, TiN/Al_2_O_3_ ≈ 350 ± 50 MW m^–2^ K^–1^, and HfN/Al_2_O_3_ ≈ 140 ± 20 MW m^–2^ K^–1^.

The results for *G* reported in Figure 3 imply that vibrational similarity between two materials has little to no effect on *G*. Many prior experimental studies have reached the opposite conclusion and reported that vibrational similarity between two materials is an important governor of thermal interface conductance.^14,30−32^ (Reference (33) is an exception to this trend and reaches conclusions about the effect of vibrational similarity that are similar to the current study.)

Prior conclusions that vibrational similarity is an important determiner of *G* are based, at least partially, on some of the following experimental results. Many of the highest reported values for *G* to date tend to be between vibrationally similar materials with strong interfacial bonds, for example, TiN/MgO,^31^ AlN/GaN,^34^ CoSi_2_/Si,^35^ SrRuO_3_/SrTiO_3_,^28^ (Al/MgO)_60GPa_,^28^ and ZnO/GaN.^36^ Alternatively, some of the lowest reported values for *G* are for interfaces between vibrationally dissimilar materials, for example, Pb/diamond,^33^ Au/Ga_2_O_3_,^37^ and Al/graphene.^38^ By compiling the results of a number of experimental studies, Giri and Hopkins^32^ and Koh et al.^39^ pointed out a positive correlation between the ratio of elastic moduli of the constituent materials and the thermal boundary conductance. A number of experimental studies have explained experimentally observed trends for *G* in metal/insulator systems as a consequence of vibrational overlap between the metal and insulator.^14,35,37^

There are key differences in the design of the current experimental study and prior work. Some of these differences make the current data set a more direct test of the effect of vibrational similarity on *G*. Many prior experimental studies report how *G* of various metal/insulator systems vary as the metal is changed.^14,18,35,38^ Changing the metal alters not only how vibrationally similar the metal and insulator are but also interfacial bonding^16,18^ and the phonon irradiance of the metal.^28^ The strength of interfacial bonds between a metal and a substrate varies, and weak interfacial bonds lead to low *G*.^16,26^ The current study tries to minimize such effects by focusing on iso-valent material systems. Another difference between the current study and prior work is that the data set in Figure 3 evaluates how *G* is affected by changes to vibrational properties of both the metal and substrate. This makes it easier to distinguish between how changes in bulk vibrational properties effect phonon irradiance^33^ versus energy transmission. We discuss the issue of phonon irradiance versus energy transmission in more detail in a later section focused on transport physics analysis.

### Effect of Unit Cell Complexity on *G*

Unit cell complexity can affect the vibrational structure of a material. With an increase in the number of atoms per unit cell, more phonon modes are in optical phonon branches, which tend to have lower group velocities. To evaluate how unit cell complexity affects interfacial transport, *G* between nitride metals and 3C-SiC, 4H-SiC, and 6H-SiC was measured. All three polytypes of SiC consist of covalently bonded Si and C atoms as building blocks, and they differ from each other only in stacking sequence. The 3C-SiC polytype has a cubic crystal lattice structure with 4 atoms in the conventional unit cell, while 4H-SiC and 6H-SiC have hexagonal lattice structures, with 8 and 12 atoms in the conventional unit cell, respectively. For both HfN and TiN, the measured interface conductances of 4H-SiC, 6H-SiC, and 3C-SiC are comparable. For TiN, *G* for TiN on SiC-6H and SiC-4H lie in the range 250–350 MW m^–2^ K^–1^, and *G* for TiN SiC-3C is ≈230 ± 30 MW m^–2^ K^–1^. For HfN, we observe that *G* for HfN/SiC-3C and HfN/SiC-6H are in the range of 170–230 MW m^–2^ K^–1^, and *G* for HfN/SiC-4H is ≈140 ± 20 MW m^–2^ K^–1^. These results indicate that increasing cell complexity of the SiC polytypes does not have a significant effect on *G*.

To date, there have been few studies of how unit cell complexity affects *G*. To our knowledge, ref (40) is the only experimental study to date that has explicitly considered the effect of unit cell complexity on *G*. In ref (40), Angeles et al. experimentally observed that accounting for unit cell complexity of yttrium-iron-garnet (YIG) crystals was necessary for agreement between experiment and theory values for *G* of Pt on YIG.^40^ Both YIG (20 atoms pre unit cell) and 6H-SiC (12 atoms per unit cell) have large basis unit cells. However, the 6H-SiC unit cell is significantly less complex than YIG. The difference between 3C-SiC versus 4H-SiC versus 6H-SiC crystals involve minor differences in stacking sequence of atomic planes along the *c*-axis. Therefore, Si and C atoms are likely to have local bonding environments similar to Si and C atoms with different basis positions.

### Effect of Interface Morphology on *G*

To determine how *G* depends on interfacial structure, a series of different TiN/AlN samples were prepared. We then studied interfacial transport between (1) sputtered TiN films and CVD-grown epilayers of AlN on sapphire, (2) sputtered TiN films and polycrystalline ceramic AlN, and (3) MBE-grown TiN film on an MBE-grown AlN layer. For the sputtered TiN deposited on CVD-grown AlN samples, the effect of AlN surface treatments prior to TiN deposition was also explored.

**Figure 4:**
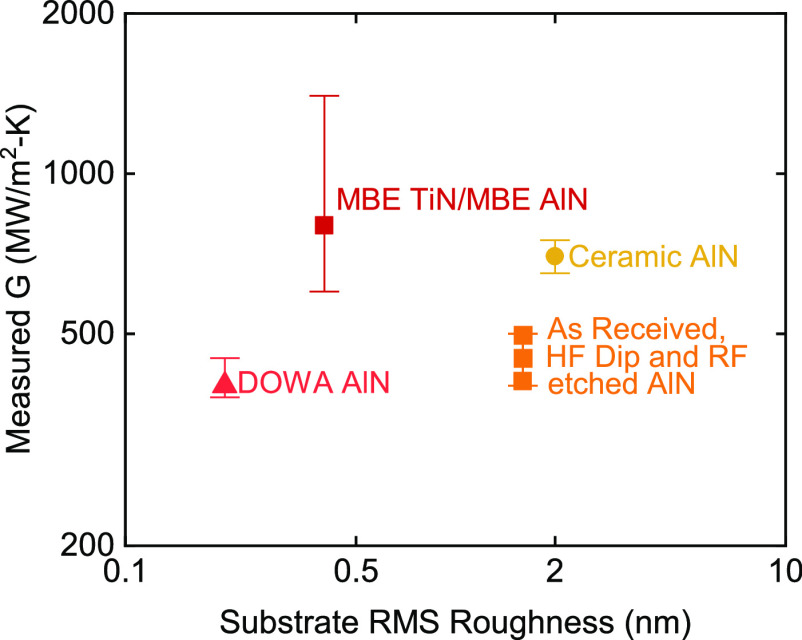
Interface conductance between TiN and various AlN crystals as a function of RMS substrate roughness. The conductance is not correlated with substrate roughness and is the highest for the epitaxial TiN/AlN sample grown via MBE.

A total of six TiN/AlN samples were studied. Details of the samples and the associated surface treatments prior to TiN deposition are reported in Table 2. Results for the interface conductance *G* as a function of substrate roughness are shown in Figure 4. For samples with films of reactively sputtered polycrystalline TiN, the AlN surface underwent one of three surface treatments: (a) untreated, (b) HF-etched for 60 s followed by immediate loading onto the high vacuum sputtering chamber, or (c) RF etched at 3.5 mTorr Ar and 35 W for 5 min.

**Table 2:** Description of Various TiN/AlN Samples Used to Study the Effect of Interfacial Disorder on *G*

sample name	stack composition	TiN growth method	AlN surface RMS roughness (nm)	AlN surface treatment	measured interface conductance (MW m^–2^ K^–1^)
MBE TiN/MBE AlN	68 nm TiN/660 nm AlN/45 nm NbN/sapphire	molecular beam epitaxy	0.4	1000 °C	800 ± 600/200
sputtered TiN/as-received AlN	40 nm TiN/380 nm AlN/sapphire	sputtering	1.6	no treatment	450 ± 50
sputtered TiN/HF AlN	40 nm TiN/380 nm AlN/sapphire	sputtering	1.6	HF Dip	450 ± 50
sputtered TiN/RF AlN	50 nm TiN/380 nm AlN/sapphire	sputtering	1.6	RF etching	450 ± 50
sputtered TiN/DOWA AlN	35 nm TiN/1000 nm AlN/sapphire	sputtering	0.24	RF etching	400 ± 50/20
sputtered TiN/polycrystalline AlN	29 nm TiN/AlN	sputtering	2	RF etching	700 ± 50

The lowest conductance was observed for TiN sputter deposited on the CVD-grown AlN epilayers. The reactive DC-sputtered TiN on CVD AlN from Kyma Technologies is ∼450 ± 50 MW m^–2^ K^–1^ for all three surface treatments. *G* for TiN on CVD AlN from DOWA is ∼400 ± 50/20 MW m^–2^ K^–1^. Therefore, we conclude that the surface treatment of the substrate does not affect the conductance of reactively sputtered TiN/AlN samples. Rather, it appears that reactive sputtering of the TiN introduces some intrinsic interfacial disorder independent of surface treatments or AlN surface morphology. The interface conductance for TiN on polycrystalline ceramic AlN ∼700 ± 50 MW m^–2^ K^–1^ is higher than the *G* for the sputtered TiN on CVD AlN epilayers. AFM showed average grain sizes of 5 nm for polycrystalline AlN. To test if grain morphology affects the TDTR response of the sample, measurements were done with a laser spot size of 1.6 nm. Small spot size measurements yielded nearly identical results for *G* as large spot size measurements. Finally, the *G* for the TiN/AlN/NbN/sapphire sample grown by molecular beam epitaxy is the highest at ∼800 MW m^–2^ K^–1^.

**Figure 5:**
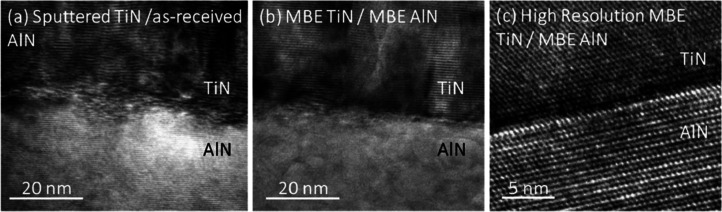
TEM micrographs of (a) 40 nm TiN reactive DC sputtered on as-received AlN and (b) 68 nm MBE-grown TiN on 660 nm MBE-grown aluminum nitride; imaged with Philips-FEI CM200 operated at 200 keV. (c) High-resolution interface image for 68 nm MBE-grown TiN on 660 nm MBE-grown aluminum nitride using Titan 80-300 aberration-corrected TEM operated at 300 keV.

Cross-sectional transmission electron micrographs were used to better understand the differences in interface morphology between the sputtered and MBE-grown TiN samples. TEM images were collected on three TiN/AlN samples. These include the MBE-grown sample, the as-received sample, and the RF-etched TiN on AlN samples. Figure 5a shows a TEM image of the TiN/AlN sample grown via sputter deposition on the untreated AlN surface. The sputtered TiN/as-received AlN shows disorderly growth of TiN layers on the AlN substrate. TEM images for TiN on RF-etched AlN were very similar. Alternatively, the MBE TiN/MBE AlN shows well-ordered TiN growth, with a clean, abrupt interface. Figure 5b,c shows TEM images for the TiN/AlN sample grown by MBE. Additional TEM images are shown in Supporting Information.

Increasing disorder at the interface between the two materials corresponds to a significant reduction of *G* from ∼800 to ∼450 MW m^–2^ K^–1^. In relative terms, the differences in conductance observed for the various TiN/AlN samples are large. However, in absolute terms, the differences in conductance are relatively small because all studied TiN/AlN interfaces have a high conductance. The sputtered TiN interfaces have an “extra” thermal resistance of only 0.75 m^2^ K/GW in comparison to the MBE sample, which is equivalent to ∼0.75 nm of an amorphous material with a thermal conductivity of 1 W m^–1^ K^–1^.

The conclusion drawn from TDTR data and analysis of TEM micrographs in our study is in accord with the existing body of knowledge. A majority of experimental studies have concluded that increase in disorder at the interface leads to a reduction in *G*. Blank and Weber^41^ showed that *G* reduces from ∼270 to ∼150 MW m^–2^ K^–1^ with an increase in interdiffusion of Si across the interface for Ti/Si systems. Mu et al.^42^ showed that reducing disorder by eliminating a 3 nm amorphous layer at the GaN/SiC interface leads to an increase in *G* from 170 m^–2^ K^–1^ to ∼230 MW m^–2^ K^–1^. Similarly, Sakata et al.^43^ showed that recrystallization of a 5 nm amorphous layer at a Si/Si interface increases *G* from ∼130 m^–2^ K^–1^ to ∼570 MW m^–2^ K^–1^. A GaN/diamond interface having good contact was shown by Yates et al.^44^ to have higher *G* (∼160 MW m^–2^ K^–1^) as compared to a GaN/diamond interface with voids (*G* ∼ 125 MW m^–2^ K^–1^). Finally, in addition to experimental evidence, theoretical models also concluded that increase in disorder at the interface reduces G.^45^

### Transport Physics Analysis

Conventional wisdom in the nanoscale heat-transfer community is that vibrational similarity between two materials effects G.^46−51^ This wisdom is premised on the assumption that interfacial heat currents are carried by phonons with vibrational frequencies that are natural to both crystals. This need for materials to be vibrationally similar to form thermally conductive interfaces poses a special challenge for ultra-wide band gap semiconductor devices. Ultra-wide band gap semiconductors such as AlN, c-BN, and diamond consist of light elements and possess strong interatomic bonds. As a result, these semiconductors have phonon frequencies much higher than most other materials.

The results for *G* between HfN and TiN and group IV and III–V semiconductors show that vibrational similarity between two materials is not required for thermally conductive interfaces. For example, TiN (Θ_D_ ≈ 600 K) is not vibrationally similar to cubic BN (Θ_D_ ≈ 1700 K) or diamond (Θ_D_ ≈ 2200 K). Not only does vibrationally similarity not appear to be required to form conductive interfaces, but dissimilarity is also not a significant impediment to interfacial heat currents. The interface formed between HfN and diamond (Θ_D_ ∼ 300 K) is just as conductive as the interface formed between HfN and Ge (Θ_D_ ∼ 360 K) or HfN and GaN (Θ_D_ ∼ 600 K).

To evaluate the relationship between *G* and bulk vibrational properties more quantitatively, kinetic gas theory can be considered. Kinetic gas theory for transport predicts that the interfacial conductance depends on the bulk vibrational properties and the probability that phonons will transmit on the interface.^52^
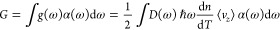
1Here *g*(ω)dω describes the phonon irradiance per Kelvin for phonons at frequency ω, and α(ω) is the frequency-dependent interfacial transmission probability of the phonons. The phonon irradiance function *g*(ω) depends on the density of states D(ω), the temperature derivative of the phonon occupation function d*n*/d*T*, the phonon energy ℏω, and the average group velocity of phonons of frequency ω in the direction perpendicular to the interface ⟨*v*_*z*_⟩. In an isotropic material, ⟨*v*_*z*_⟩ equals *v*_*z*_/2.

Theoretical models, such as the acoustic and diffuse mismatch model, assume that α(ω) depends on the vibrational similarity of the two materials forming the interface. If all the energy from material 1 impinging on the interface is carried by phonons with frequencies that atoms of material 2 do not naturally oscillate at, then α(ω) is expected to be small or zero. With this in mind, we define two terms that allow us to quantify what the experimental data in Figure 3 implies about the relationship between α(ω) and vibrational similarity. We define the average phonon transmission probability for an interface as
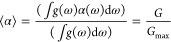
2Here, *G* is the experimentally measured interface conductance between two materials, for example, *G* = 500 MW m^–2^ K^–1^ for TiN/diamond. *G*_max_ is the maximum conductance for the interfaces, as calculated in eq 1. Due to requirements for detailed balance, *G*_max_ for an interface is limited by whichever of the two materials is vibrationally softer. For example, in the case of TiN/diamond, *G*_max_ = 1200 MW m^–2^ K^–1^ = {*G*_max_}_TiN_.

We define vibrational similarity as the inner product of the phonon irradiance functions of the two materials
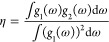
3

Here, material “1” is the nitride metal and material “2” is the substrate.

Physically, η describes the similarity in the spectral distribution of the phonon irradiance per Kelvin of the two materials. The value of η approaches unity if heat in both materials is carried by phonons with similar frequencies and approaches zero if heat is carried by phonons of different frequencies. η is evaluated for each material system studied by assuming an isotropic quadratic dispersion relationship (see Supporting Information). For simplicity, it is assumed that heat currents are carried by acoustic branches and optic modes are neglected.

**Figure 6:**
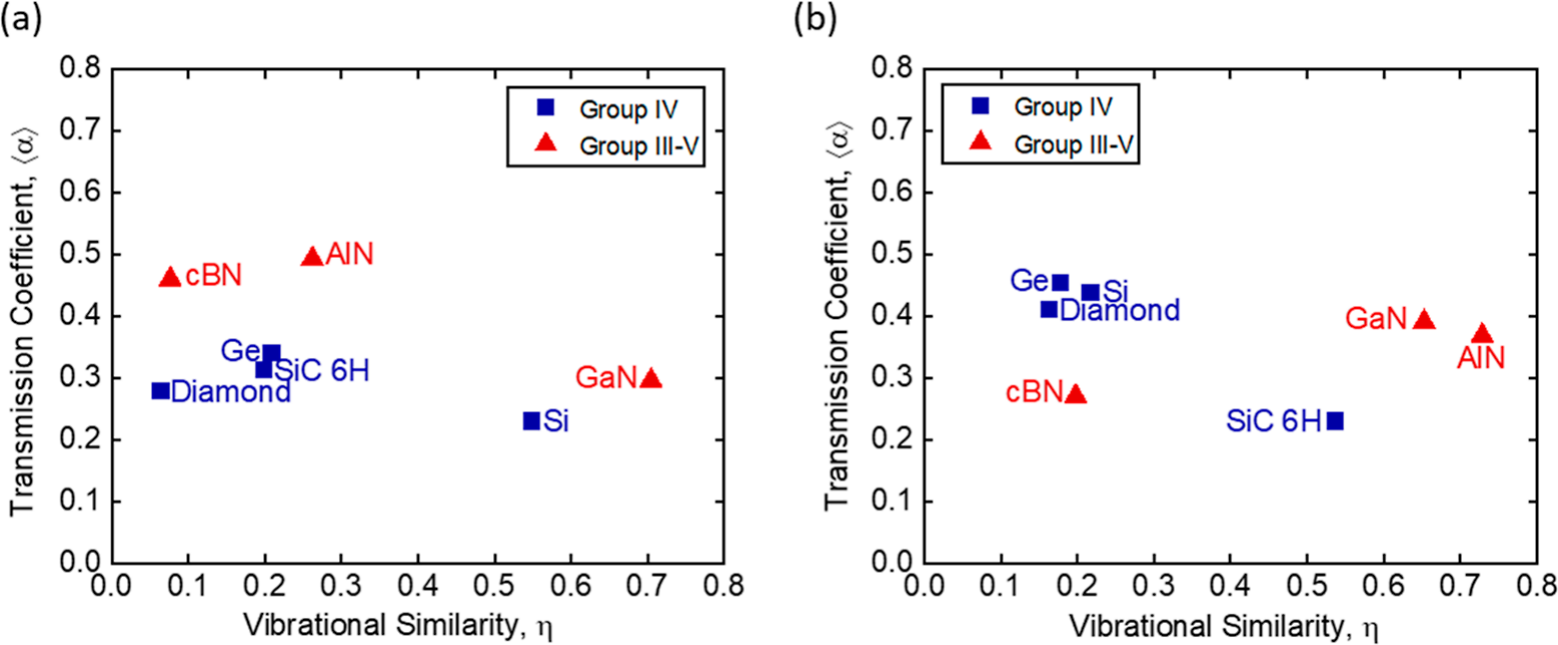
Transmission coefficient ⟨α⟩ vs the vibrational similarity η between (a) HfN and (b) TiN and group IV and III–V substrates. ⟨α⟩ is the ratio of interface conductance value derived from the experimental data in Figure 3 and the theoretical maximum conductance of the softer of the two materials forming the interface; see eq 2. η is a theoretically calculated property for each material combination; see eq 3. η and ⟨α⟩ are not correlated with one another in either the TiN or HfN system.

Figure 6a,b shows the relationship between the average transmission coefficient ⟨α⟩ and the vibrational similarity η for HfN and TiN with the semiconductor crystals. The trends for both groups do not show any dependence of ⟨α⟩ on the vibrational similarity between the two materials forming the interface. For example, the value for ⟨α⟩for HfN/diamond ≈0.3 is similar to ⟨α⟩for HfN/Si, even though Si is approximately six times more vibrationally similar to HfN than to diamond. Therefore, we conclude that the vibrational similarity between materials has only a minor impact on interfacial thermal transport properties in these systems.

**Figure 7:**
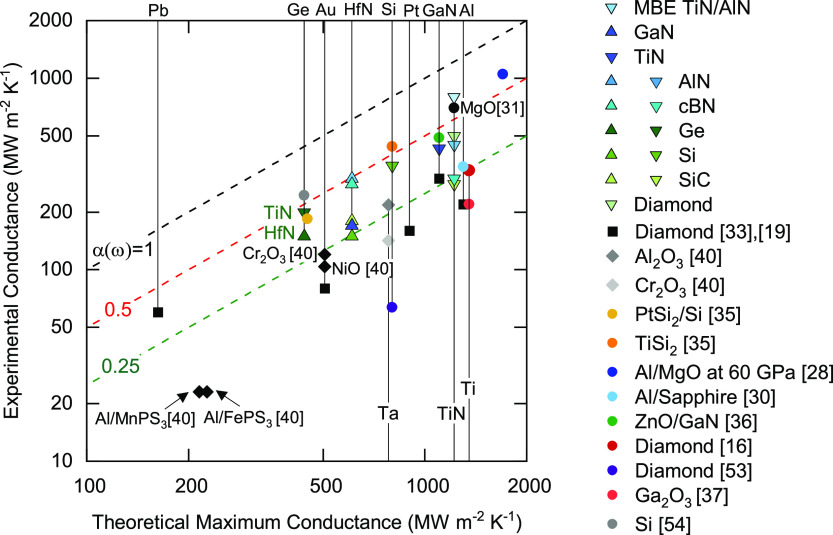
Measured interface conductance *G* vs theoretical maximum conductance *G*_max_ for different material systems. The theoretical maximum conductance corresponds to the limit where the probability of phonon transmission at the interface is unity, α(ω) = 1. Green, red, and black dashed lines represent predictions for the conductance when the average transmission probability α(ω) = 0.25, 0.5, and 1, respectively. The data represented by triangle symbols are from the current study. The drop lines and the corresponding labels indicate the *G*_max_ of the softer material forming the interface, which determines the theoretical maximum conductance for the material system.

The average transmission coefficient ⟨α⟩, a property that is estimated using the vibrational properties of the vibrationally soft material, varies between a relatively narrow range of ∼0.3 and 0.5 in most systems (Figure 6). This result implies that interface conductance depends strongly on the vibrational properties of the vibrationally soft material. To reiterate this, the results are compared with the existing body of knowledge for interface conductance values across different material systems. Figure 7 shows *G* versus theoretical maximum conductance for that material system, that is, *G*_max_. The results of this study are compared to literature data for metal/diamond systems at high pressures,^33^ metal/oxide and metal/trisulfide systems,^40^ silicide/Si systems,^35^ epitaxial TiN/MgO,^31^ Al/MgO at 60 GPa,^28^ Al/Sapphire,^30^ ZnO/GaN,^36^ Ti/Ga_2_O_3_,^37^ Ti/diamond,^16^ GaN/diamond,^19^ diamond/Si,^53^ and Si/Ge.^54^ In all these systems, the measured interface conductance values are correlated with the theoretical maximum *G*_max_. Consistent with the results of this study, most of the experimental data compared to in Figure 7 are consistent with average interfacial transmission probabilities between 0.25 and 0.5.

Another noteworthy fact illustrated in Figure 7 is that the TiN thermal conductances reported in this study are quite high in comparison to typical values.^16^ To our knowledge, the interface conductance of 500 MW m^–2^ K^–1^ between TiN and diamond is the highest thermal conductance ever reported for any diamond system.^16,33^ The interface conductance of 800 MW m^–2^ K^–1^ reported for TiN/AlN is among the highest reported values for any interface for which the heat is carried by phonons.^31^

The effect of bulk vibrational properties and vibrational similarity *G* is often discussed in terms of elastic versus inelastic processes.^30,31,35,37,39,55^ For vibrationally dissimilar materials, the phase space for elastic interfacial scattering is small because of limited vibrational overlap.^52^ Therefore, in dissimilar materials, it is generally agreed that energy transmission occurs primarily via inelastic processes.^33,47^ How important inelastic processes are for energy transport at interfaces between vibrationally similar materials is an active area of research. Experimental studies of *G* between vibrationally similar materials often conclude that energy is transmitted via elastic processes because data are in reasonable agreement with kinetic gas theory models that assume phonons scattering elastically at interfaces.^30,31,35^ However, recent advances in theoretical methods for studying interfacial heat transfer suggest inelastic processes are important in both vibrationally similar and dissimilar material systems.^56−59^ For example, theoretical models based on Green’s functions^56,57^ and molecular dynamics^58^ both predict that ∼50% of heat is carried across interfaces between vibrationally similar Si and Ge via inelastic processes. The results in Figure 6 and 1 are consistent with these recent theoretical predictions that inelastic processes play an important role in both vibrationally similar and dissimilar material systems. We observe that energy transmission rates are similar for both vibrationally similar and dissimilar materials (Figure 6). Similar energy transmission rates suggest that energy transport occurs via similar mechanisms in both types of systems.

Recent theoretical and experimental research have explored the existence of interfacial modes and their effect on interfacial heat transfer. Interfacial modes could mediate energy transfer between bulk vibrational modes with different frequencies.^57^ High-energy resolution electron energy loss spectroscopy studies in scanning transmission electron microscopy have confirmed the presence of interfacial phonon modes.^54,60,61^ Theoretical methods capable of spectrally resolving the interfacial heat current suggest that interfacial modes play an important role.^58^ It is possible that interfacial modes are partly responsible for why energy transmission rates are similar in both vibrationally similar and dissimilar materials (Figure 6). However, the mechanisms by which energy is transmitted across the interface do not alter the results of our study. The results in Figure 3 and 6 reveal how much of the energy that impinges upon an interface gets transmitted, but not by which mechanism it does so, whether by harmonic processes, anharmonic processes, or by interfacial modes.

### Effect of Improvement in *G* on Device Temperature Rise

Finally, the implications of this study for thermal management of wide and ultra-wide band gap devices are briefly considered. For electronic devices, the relevant interfaces are between semiconductors, for example, GaN/diamond or AlN/SiC. However, TiN has similar vibrational properties as GaN and AlN. Therefore, this study benchmarks what interface conductance values are likely to be possible between wide-band gap device heterostructures and high thermal conductivity substrates such as diamond, SiC, AlN, and c-BN. Observed values for the interface conductance between HEMT-heterostructures and diamond range from refs (19) and (62), 10 to 300 MW m^–2^ K^–1^. To quantify how thermal performance of a device will be affected by increasing *G* to values similar to the ones reported in Figure 3, that is, *G* ≈ 500 MW m^–2^ K^–1^, a device architecture like the one reported in ref (63) is considered. This device has 2 × 0.7 mm^2^ rectangular transistors with 22 gate fingers of 0.5 × 150 μm^2^ on a stack of 20 nm AlGaN on 0.5 μm GaN on a 100 μm diamond substrate. The maximum temperature of the device is calculated as a function of boundary resistance between the device multilayer and diamond substrate. Assuming temperature-independent thermal properties, Δ*T*_device_ = *J*/*R*_eff_. The effective thermal resistance *R*_eff_ ≈ *R*_interface_ + *R*_other_. Here, *R*_interface_ is the thermal boundary resistance between the device multilayer and the diamond substrate. *R*_other_ includes thermal resistance from the device layer, the diamond substrate, die-attached layer, and the convective boundary at the bottom of the die. See Supporting Information for more details of model for thermal performance of the device. This model implies that, for *G* ≈ 10 MW m^–2^ K^–1^ between the device multilayer and diamond, the temperature rise for a power density of 10 W mm^–1^ is ∼2000 K. The power density of an HEMT is defined as the total power per unit gate width of the device. Alternatively, with a more typical boundary conductance value of ∼100 MW m^–2^ K^–1^, the device temperature rise will be ∼350 K. Finally, for a conductance comparable to the ones reported in Figure 3, that is, *G* ≈ 500 MW m^–2^ K^–1^, the maximum temperature rise of the device would be ∼200 K.

## Conclusions

The dependence of *G* on vibrational similarity of the materials forming the interface, complexity of unit cells, and interfacial structure has been studied by depositing TiN and HfN on group IV and group III–V crystals. *G* for HfN on group IV and III–V materials ranged from 140 to 300 MW m^–2^ K^–1^, whereas *G* for TiN on group IV and III–V materials ranged from 200 to 800 MW m^–2^ K^–1^. There was no evidence that *G* depended on whether the materials forming the interface were vibrationally similar. Instead, it was found that *G* depended on the vibrational properties of the vibrationally soft material as well as the interfacial structure. This study establishes what material properties govern thermal transport at interfaces with wide- and ultra-wide band gap materials. These fundamental findings should be useful in ongoing efforts to optimize thermal transport in high power electronic devices.

## Supporting Information Available

Atomic force microscopy scans; metal thickness and thermal conductivity analysis; Raman spectroscopy; transmission electron micrographs; *G*_max_ calculation parameters; and modeling of the thermal performance of an HEMT device on diamond (PDF)

## Abbreviations

TDTR: time-domain thermoreflectance
PAMBE: plasma-assisted molecular beam epitaxy
EIES: electron impact emission spectroscopy
MBE: molecular beam epitaxy
AFM: atomic force microscopy
TEM: transmission electron microscopy
CVD: chemical vapor deposition
RF: radio frequency
