# Supplementary material for: Properties for Thermally Conductive Interfaces with Wide Band Gap Materials
Source: ACS Appl Mater Interfaces. 2022 Jul 27;14(31):36178–88. doi: 10.1021/acsami.2c01351 (PMC9376929; doi:10.1021/acsami.2c01351)
Supplement: Supplementary file 1 — am2c01351_si_001.pdf [file am2c01351_si_001.pdf]

Supporting Information

Properties for Thermally Conductive Interfaces with Wide-Bandgap Materials

*Samreen Khan[a], Frank Angeles[a], John Wright[b], Saurabh Vishwakarma[c], Victor H. Ortiz[a], Erick Guzman[a], Fariborz Kargar[a], Alexander A. Balandin[a], David J. Smith[c], Debdeep Jena[b], H. Grace Xing[b], Richard Wilson[a,]**

[a] University of California Riverside, Riverside, California 92521, United States

[b] Cornell University, Ithaca, New York 14850, USA

[c] Arizona State University, Tempe, Arizona 85287, United States

* Corresponding author: E-mail: rwilson@ucr.edu

1. Atomic Force Microscopy Scans

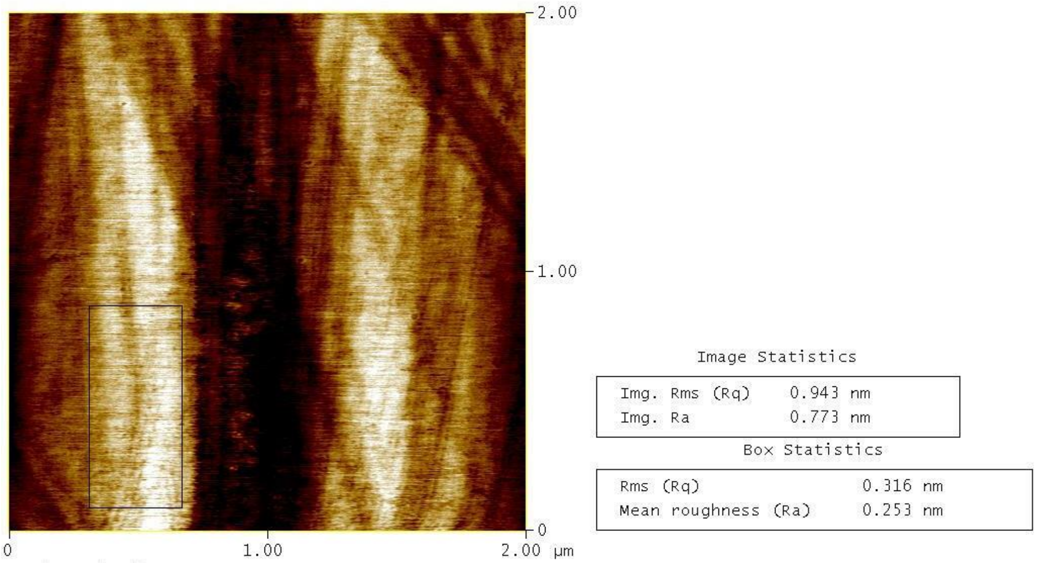

Figure S1. AFM scan on 2 µm x 2 µm area of the Diamond sample with (100) orientation purchased from Element Six. RMS roughness observed ≈1 nm.

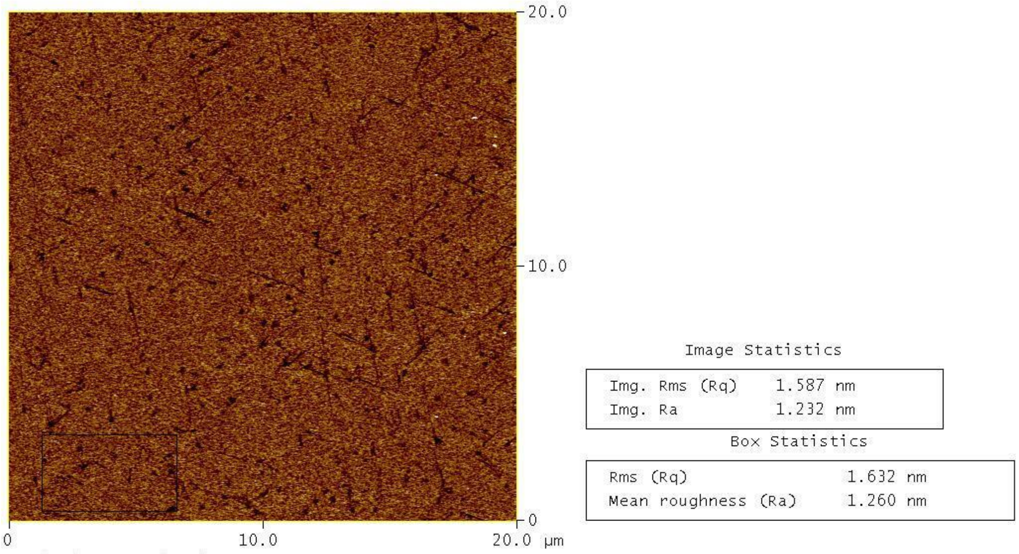

Figure S2. AFM scan on 20 µm x 20 µm of the CVD grown AlN substrate purchased from Kyma Technologies. RMS roughness observed ≈1.6 nm.

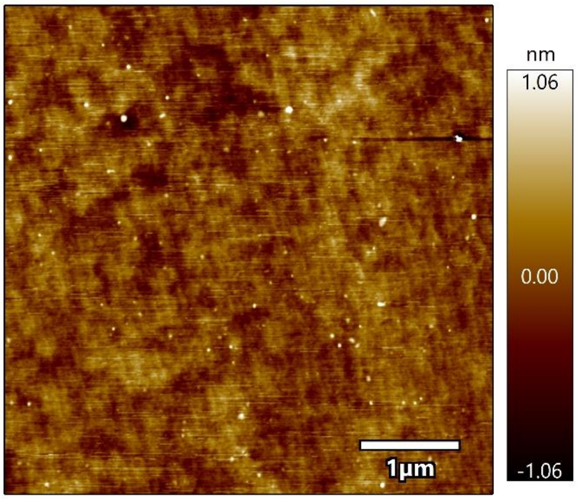

Figure S3. AFM scan performed at Cornell University on 5 µm x 5 µm of the CVD grown AlN substrate purchased from DOWA. RMS roughness observed ≈0.24 nm.

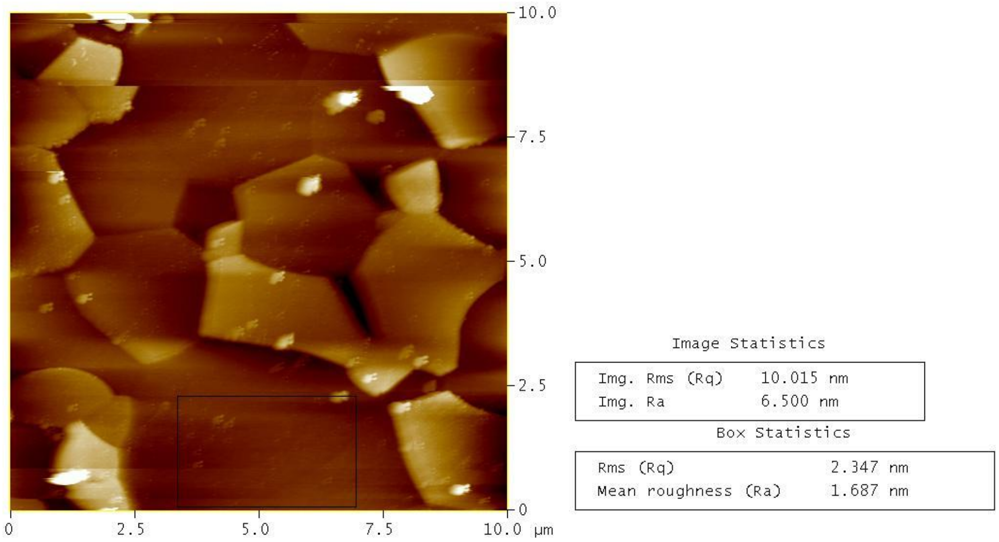

Figure S4. AFM scan of Polycrystal ceramic AlN from University wafer. We observed an RMS roughness of ≈2 nm on 5 µm x 5 µm of the substrate.

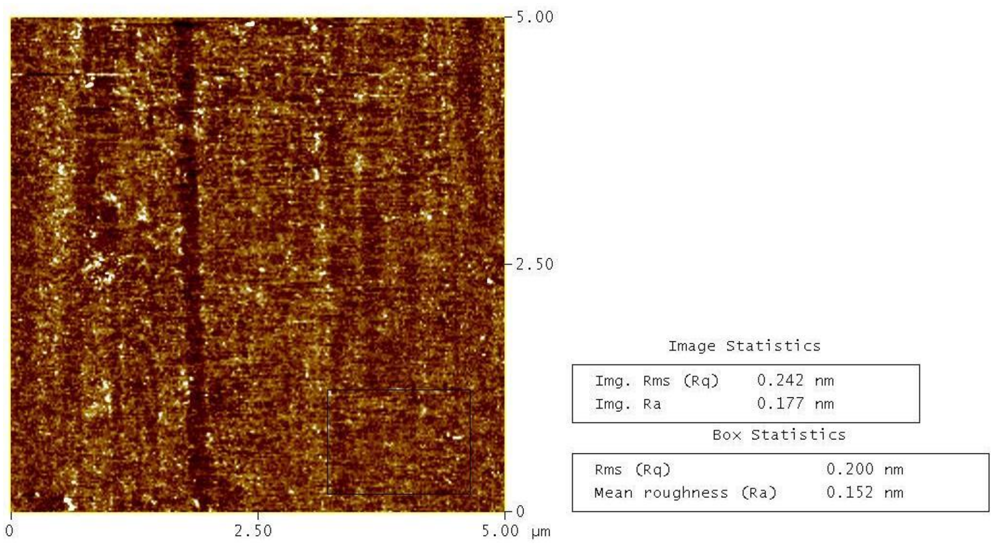

Figure S5. AFM scan on 5 µm x 5 µm of the (0001) Al_2O_3 substrates from University Wafers. RMS roughness observed ≈2 nm.

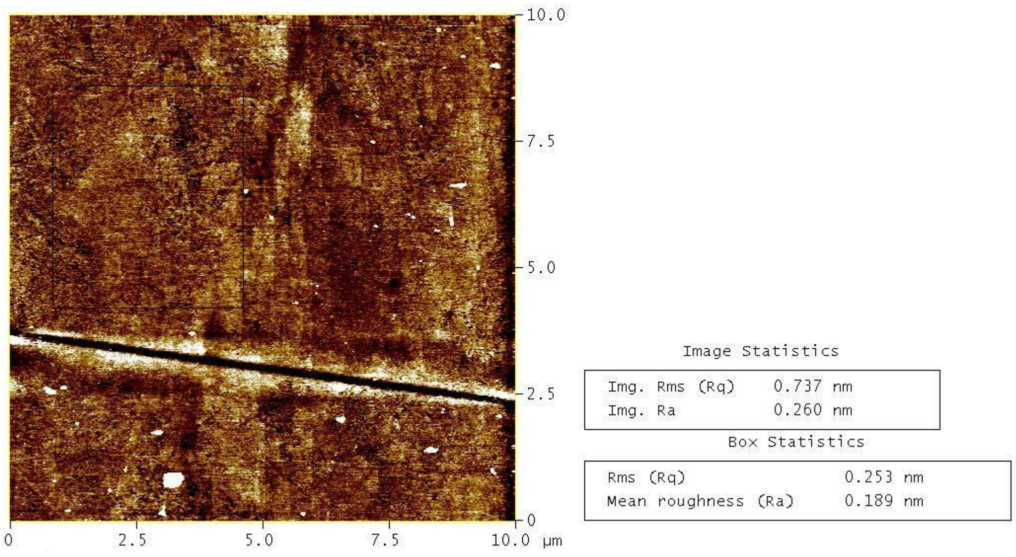

Figure S6. AFM scan on 10 µm x 10 µm of the TiN on (100) MgO from MSE Supplies. RMS roughness observed ≈0.2 nm.

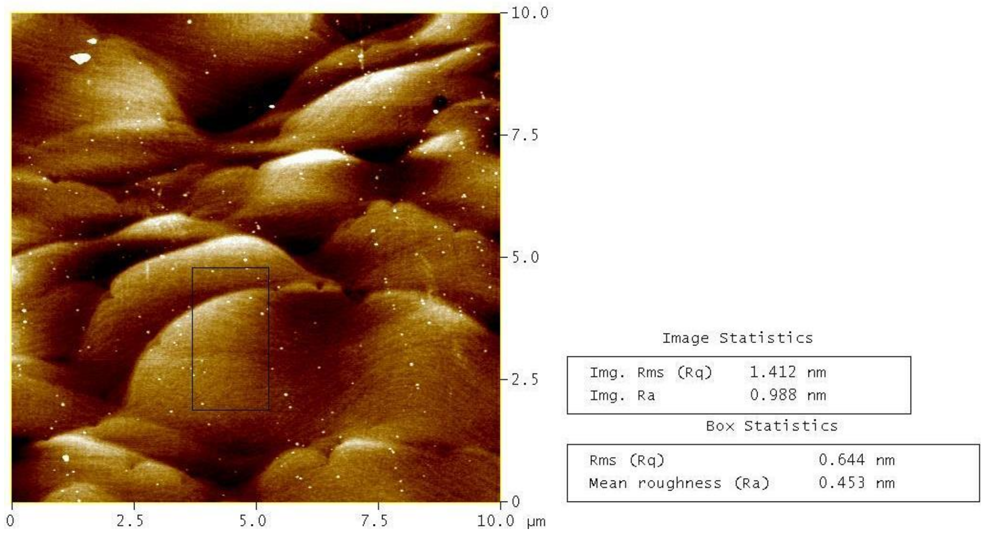

Figure S7. AFM scan on 10 µm x 10 µm of the TiN on (0001) GaN substrates were from MTI Corporation. RMS roughness observed ≈1 nm.

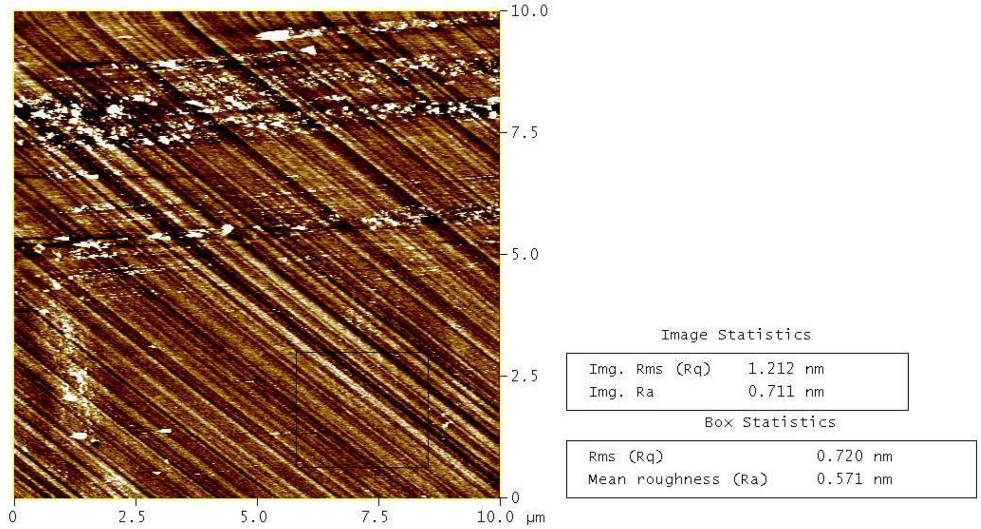

Figure S8. AFM scan on 10 µm x 10 µm of the TiN on high-purity element-six ELSC grade {100} diamond from Element Six. RMS roughness observed ≈0.7 nm.

2. Metal Thickness and Thermal Conductivity Analysis

The thicknesses of the nitride metal layers were determined in two ways. First, by picosecond acoustics observed on a control sample of nitride metal/SiO_2/Si stack[1]. The control samples were deposited at the same time as the samples of interest. To interpret picosecond acoustic signals, we assume average longitudinal speeds of sound in TiN and HfN to be ~10 nm/ps [2] and ~5.5 nm/ps. We derived the value for the speed of sound in HfN from elastic constant data [3]. We also measured film thickness by treating it as a fit parameter when analyzing TDTR data collected on control samples. For control samples, e.g. TiN/sapphire or TiN/SiO_2, all thermal model parameters are well known other than metal thickness. Both methods for measuring thickness provide values within 10% of each other.

To determine the thermal conductivity of TiN and HfN, we prepared a 200 nm HfN/sapphire and 200nm TiN/sapphire samples. We did front-back TDTR measurements on 200nm TiN/Sapphire and 200nm HfN/ Sapphire samples. These TDTR experiments yield thermal conductivities for our TiN and HfN films of 26 and 14 W m^{-1} K^{-1}. The thermal conductivity Λ of the nitride films includes both electron and phonon contributions, $\Lambda = \Lambda_{phonon} + \Lambda_{electron}$. Using four-point probe measurements of the electrical resistivity and the WF-law, in these 200 nm thick samples we estimate $\Lambda_{electron} \approx$ 10 and 4 W m^{-1} K^{-1}. Therefore, Λ_{phonon} of TiN and HfN is $\approx$16 and $\approx$10 W m^{-1} K^{-1}, respectively.

The TiN and HfN films deposited on different substrates have different electrical conductivities, likely due to differences in the polycrystalline grain sizes. When analyzing our TDTR data, we account for variation in electrical transport by using the WF-law to predict $\Lambda_{electron}$ for each sample, see Table 1. We assume phonon thermal conductivities are sample independent. This

latter assumption may not be rigorously valid. However, the sensitivity of our measurements to Λ of the nitride-metal is small.

Table S1: $\Lambda_{electron}$ for the nitride metals deposited on the group IV and III-V samples.

Sample	Resistivity 10^{-6} (Ω m)	$\Lambda_{electron}$ (W m^{-1} K^{-1})
TiN/Diamond	0.7	9.5
TiN/SiC 4H	0.8	9
TiN/SiC 6H	0.5	13
TiN/SiC 3C	0.7	9.4
TiN/Si	0.5	15
TiN/Ge	1.1	6
TiN/AlN (as received)	1.3	5.6
TiN/AlN (RF etched)	0.4	18
TiN/AlN (HF dipped)	1.4	5.1
TiN/AlN (DOWA)	0.3	22
TiN/GaN	0.3	25
HfN/Diamond	1.7	4.2
HfN/SiC 4H	0.4	17
HfN/SiC 6H	0.3	20
HfN/SiC 3C	0.9	7.9
HfN/Si	1.2	5.9
HfN/Ge	1.2	5
HfN/AlN	0.8	8
HfN/GaN	1.2	6

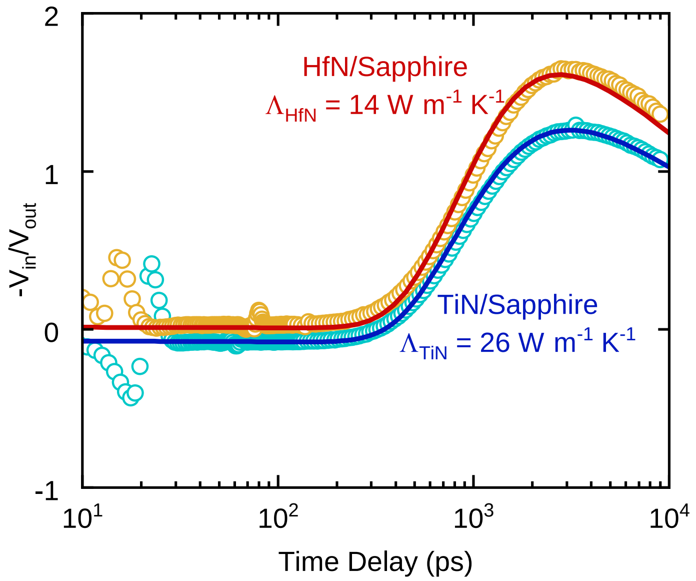

Figure S9. The yellow and blue points are the data collected from TDTR measurement of 200 nm HfN/Sapphire and 200 nm TiN/Sapphire, respectively. The solid red and blue curves are the prediction of the thermal model for the two samples. The thermal model agrees with the data at a thermal conductivity of 26 m^{-1} K^{-1} for TiN/Sapphire and at 14 W m^{-1} K^{-1} for HfN/Sapphire.

Uncertainty in the total thermal conductivity of the sputtered nitride metal films was of concern to us. For our measurements of the interface conductance to be as accurate as possible, we want the thermal resistance of the metal film to be small. If the thermal resistance of the metal film is small, the temperature evolution of the sample surface will be governed by the interface conductance. In our initial experiments, we deposited TiN films with a thickness of 55-60 nm. But we found that for samples with high G, our TDTR signals had a small amount of sensitivity to the thermal conductivity of the nitride, which led to larger error bars for G. To eliminate sensitivity to the TiN thermal conductivity, we prepared samples with thinner TiN layers. We found preparing samples with a TiN layer with a thickness between 30 and 40 nm effectively eliminates sensitivity

to the TiN's thermal conductivity, see Supplemental Figures 10(a) and 10(b). For HfN samples, since the thermal conductance is lower than for the TiN material systems, a thin HfN transducer is not necessary, see Supplementary Figure 10(c).

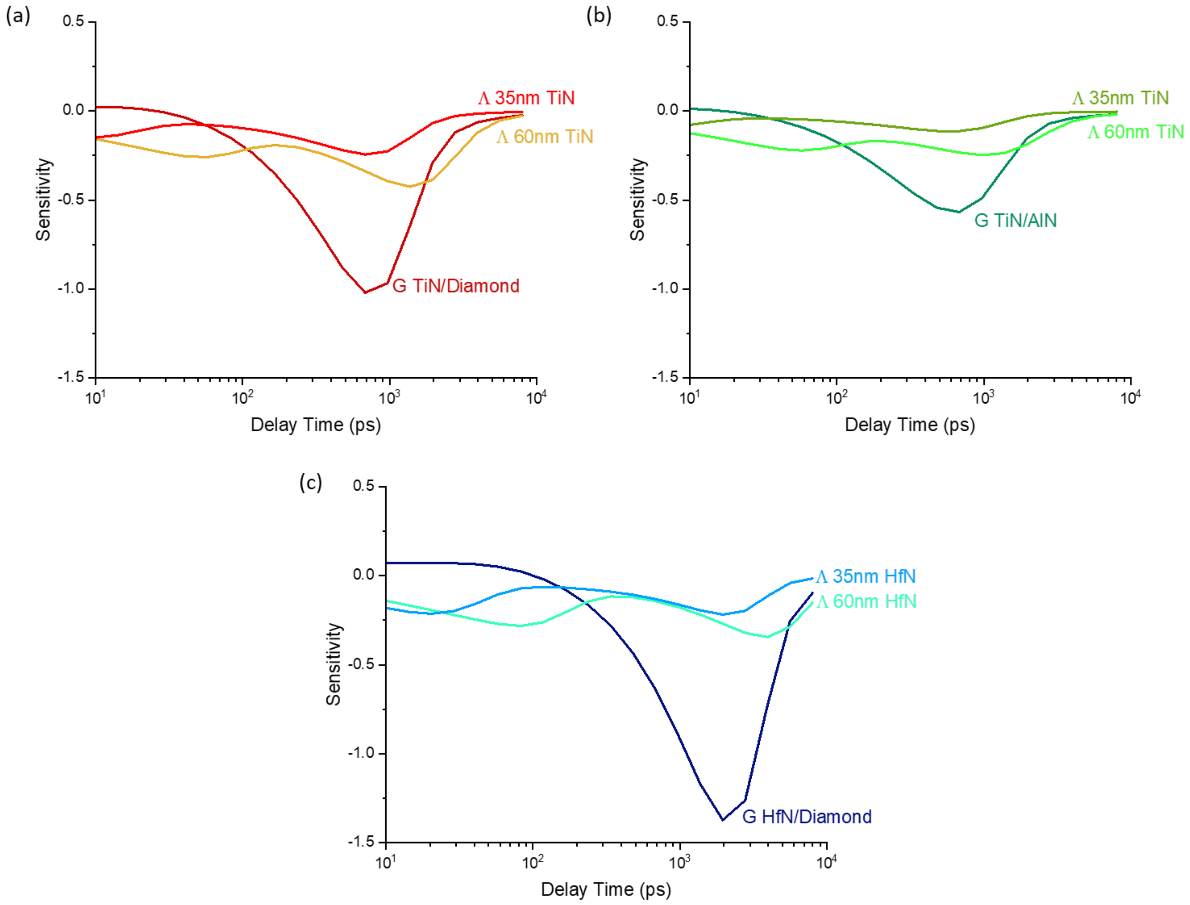

Figure S10. The calculated sensitivity curves for (a) TiN/Diamond with G = 500 MW m^{-2} K^{-1}, Λ_{TiN} = 20 W m^{-1} K^{-1} (solid line) and Λ_{TiN} = 10 W m^{-1} K^{-1} (dashed line); (b) AlN/Diamond G = 450 MW m^{-2} K^{-1}, Λ_{TiN} = 20 W m^{-1} K^{-1} (solid line) and Λ_{TiN} = 10 W m^{-1} K^{-1}; and (c) HfN/Diamond with Λ_{HfN} = 14 W m^{-1} K^{-1} and G = 170 MW m^{-2} K^{-1}.

3. Substrate Thermal Conductivity

To determine the thermal conductivity of the bulk substrates, we performed TDTR on SiC 4H, SiC 6H, Si, Ge and ceramic AlN with ~80 nm of Al as the transducer. We used picosecond acoustic analysis to determine the thickness of the DC sputtered aluminum film, with an uncertainty of 5%. The thermal conductivity and heat capacity of Al, heat capacities of the substrates[4–14] and thicknesses of the constituent layers were the input parameters for the thermal model. We fit the thermal model to the TDTR data by using the thermal conductivity of the substrate as the fitting parameter. Table S2 lists the best-fit values of the thermal conductivity of the substrates. The error bars indicate the range of thermal conductivity values within 5% RMS error due to the uncertainty in the thickness of the aluminum film. These measured substrate thermal conductivities were then used as input parameters for nitride metal/bulk substrate (SiC 4H, SiC 6H, Si, Ge and ceramic AlN) data. Overall, the TDTR measured thermal conductivities are in good agreement with literature values.

Table S2: Best fit values for the thermal conductivity of substrates

Sample	Λ Measured ($W\ m^{-1}\ K^{-1}$)	Λ Literature ($W\ m^{-1}\ K^{-1}$)
Diamond	2200 ± 100	600 – 2500 [15]
SiC 4H	350+/- 70/30	350 [16]
SiC 6H	340+/- 70/30	300 [16]
Si	145 ± 15	145 [17]
Ge	57 +/- 18/9	58 [18]
cBN	300 - 450	870 [19]
Ceramic AlN	180 ± 20	17 – 285 [20]

For TiN/Diamond and TiN/cBN samples, the thermal conductivity of the diamond substrate and cBN crystals was used as a fit parameter along with the interface conductance values. It was possible to have two fit parameters since the model is sensitive to the thermal conductivity of the substrate and the interface conductance at different time delays, see Figure S11. The thickness of the TiN transducer was determined using picosecond acoustic analysis with 5% error. The error bars for diamond thermal conductivity show the range of thermal conductivity values for RMS error ≤ 10% due to the uncertainty in the thickness of the TiN layer. We performed TDTR on multiple cBN crystals and measured thermal conductivity values between 300-450 W m^{-1} K^{-1}. The thermal conductivity we observe for c-BN is lower than has been reported for high quality c-BN single crystals. This is not surprising, as the crystals we measure have a yellowish tint. Since c-BN has a band-gap wider than visible light, a yellowish tint indicates a non-trivial concentration of point-defects.

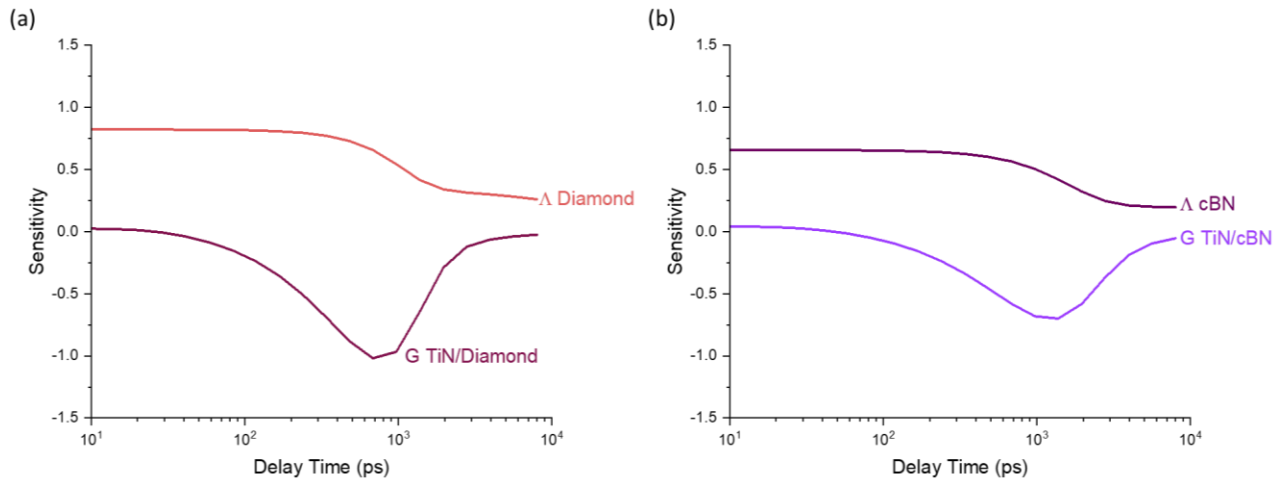

Figure S11. The calculated sensitivity curves for (a) TiN/Diamond with G = 500 MW m^{-2} K^{-1}, $\Lambda_{Diamond}$ = 2200 W m^{-1} K^{-1}; (b) TiN/cBN with G = 330 MW m^{-2} K^{-1}, Λ_{cBN} = 350 W m^{-1} K^{-1}.

For SiC-3C film on (100) Si and GaN film on (0001) Sapphire from MTI Corporation, the thermal conductivity of the thermally thick films was used as a fit parameter along with the interface conductance for TiN/SiC 3C and TiN/GaN samples. This was possible since the model is sensitive to the thermal conductivity of the film and the interface conductance at different time delays, allowing us to fit for both parameters, see Figure S12. We measured the thermal conductivity of GaN and SiC 3C to be 100 ± 10 W m^{-1} K^{-1} and 90 ± 20 W m^{-1} K^{-1}, respectively. The measured thermal conductivities for GaN and SiC 3C are only ~ 50% and ~ 25% of bulk thermal conductivity values, respectively. Epi-layer films often have lower thermal conductivity than bulk single crystals due to defects, e.g. dislocations.

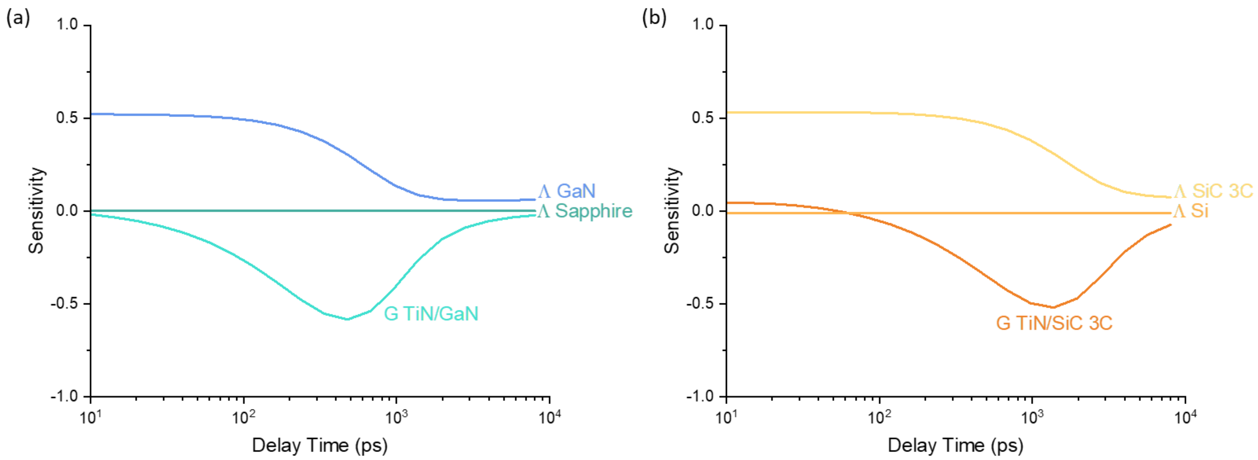

Figure S12. The calculated sensitivity curves for (a) TiN/GaN/Sapphire with G = 330 MW m^{-2} K^{-1}; (b) TiN/SiC 3C/Si with G = 230 MW m^{-2} K^{-1}.

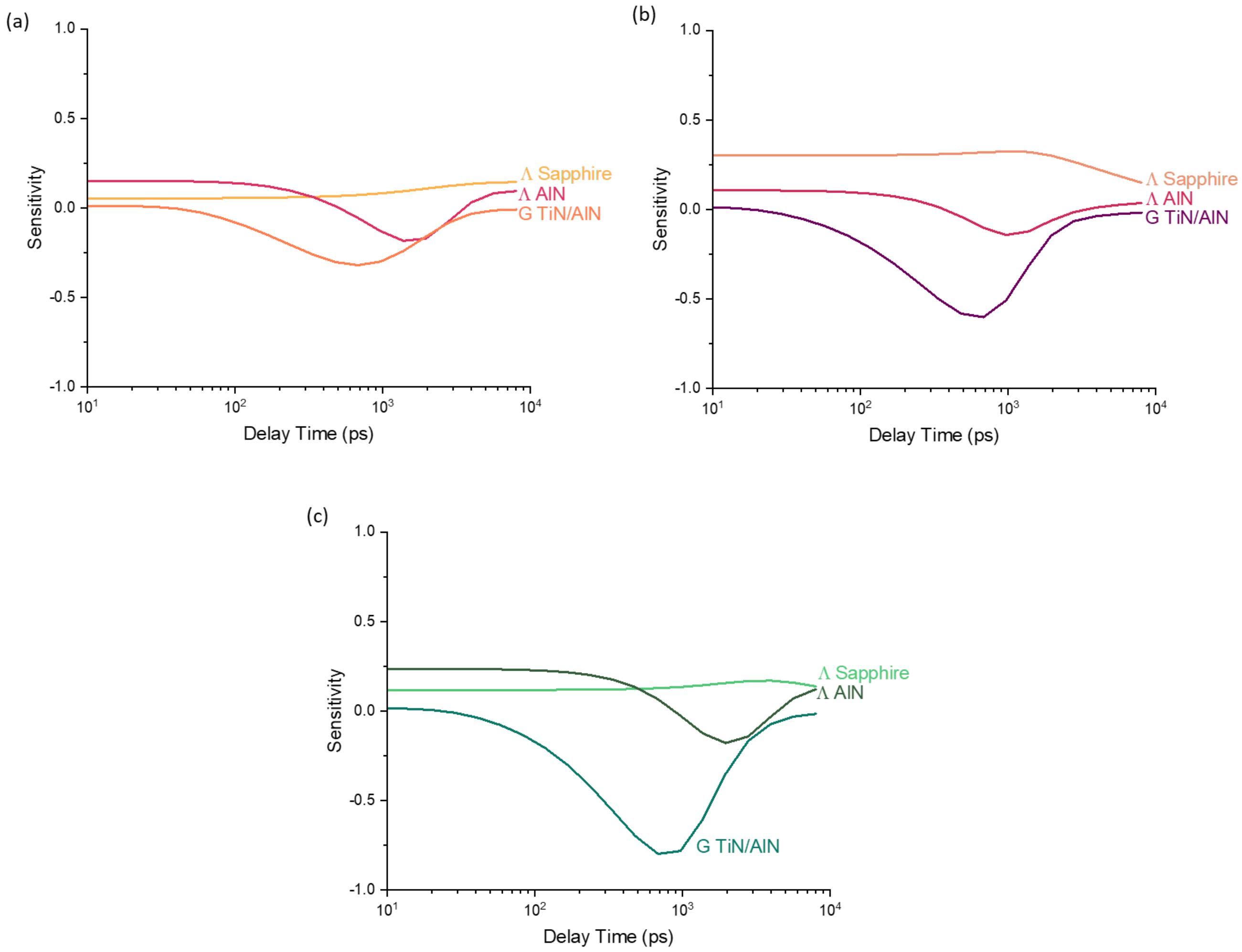

Figure S13. The calculated sensitivity curves for (a) MBE TiN/MBE AlN with G = 330 MW m^{-2} K^{-1}; (b) TiN/Kyma AlN with G = 450 MW m^{-2} K^{-1}; (c) TiN/DOWA AlN with G = 400 MW m^{-2} K^{-1}.

Finally, the sensitivity plots in Figure S13 indicate that the thermal model is not sensitive to the thermal conductivities of the thin-film AlN for all three substrates: CVD AlN epilayers from DOWA, CVD AlN epilayers from Kyma Technologies and MBE grown AlN from Cornell University. This is because AlN has high thermal conductivity and the AlN films are not thick enough for effects of its thermal resistance to be measured at picosecond time-scales. Since the

sensitivity to the thin-film AlN is low, it is not possible to accurately fit for the thermal conductivities of these films. Due to the low sensitivity however, our best-fit values for the interface conductance for all three samples does not depend on the thermal conductivity of the AlN thin-films.

4. Raman Spectroscopy

Raman spectroscopy (Renishaw inVia) measurements were performed on HfN and TiN deposited on Si and MgO substrates in the backscattering configuration using 633 nm (red) laser excitation wavelength. [1] The cutoff frequency is 110 cm^{-1}. The results are shown in Figure S14 and Figure S15. In both plots, the black and blue curves correspond to the Raman spectra of bare Si and MgO substrates, respectively. Silicon has only one Raman-active mode at ~520 cm^{-1} which is attributed to the LO/TO phonon polarization branches[21].The additional peaks are originated due to the relaxation of Raman selection rules as a result of doping or presence of defects in Si[22]. MgO, HfN, and TiN have rock-salt crystal structures in which 1st-order Raman peaks are forbidden by symmetry[23]. As seen in Figure S14 and Figure S15, the spectra collected from the pristine MgO substrate (blue curves) do not exhibit any peaks confirming that the crystal is almost defect free. In case of HfN/Si and HfN/MgO structures (Figure S14, red and green curves), a broad peak is observed at ~170 cm^{-1}. The peak is attributed to the first-order acoustic bands activated by the presence of nitrogen vacancy in HfN[23]. In case of TiN, no Raman peaks are detected in the shown frequency range. The latter confirms the deposition of stochiometric TiN on both substrates.

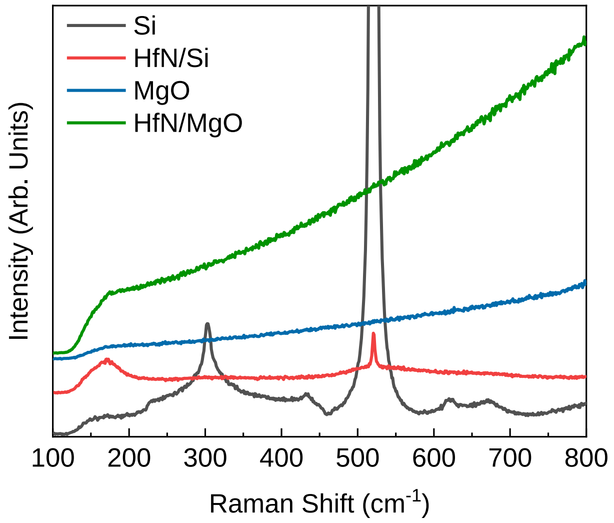

Figure S14. Raman peaks of HfN on MgO and Si. The broad peaks observed at ~170 cm^{-1} is attributed to HfN acoustic bands activated by nitrogen deficiency.

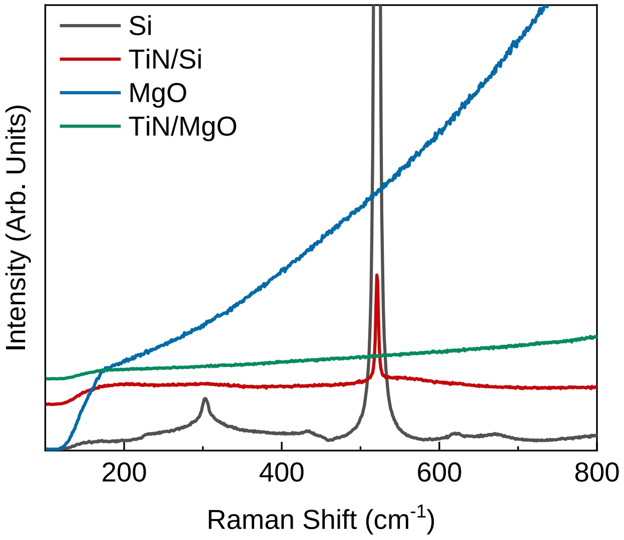

Figure S15. Raman peaks of TiN on MgO and Si. The absence of1st-order Raman peaks in TiN heterostructures confirms the deposition of stochiometric TiN on both Si and MgO substrates.

5. Transmission Electron Micrographs

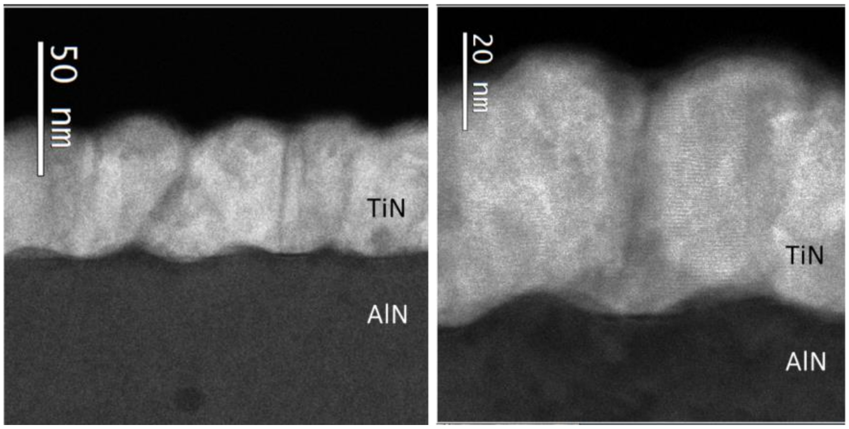

Figure S16. High-angle annular dark-field images of 50 nm DC sputtered TiN on RF etched AlN from Kyma Technologies. These images were taken using probe-corrected ARM200F scanning TEM operated at 200 keV. TiN film is granular on the 15-20nm scale and there is minimal evidence of epitaxial growth.

6. Isotropic Quadratic Dispersion Relation

We assumed a simple isotropic quadratic dispersion relationship to evaluate the phonon irradiance per Kelvin for TiN, HfN, group IV materials and III-V materials.

$$\omega(k) = v_g k \tag{1}$$

Here $\omega(k)$ is the phonon density of states, v_g is the phonon group velocity and k is the wave vector.

$$v_g = v_s - 2bk \tag{2}$$

Here v_s is the sound velocity in the material and b is a constant,

$$b = \frac{v_s\, q_D\, \omega(\mathrm{k})_{max}}{{q_D}^2} \tag{3}$$

q_D is the reduced Debye wave vector,

$$q_D = (6\pi^2 n)^{1/3} \quad (4)$$

$$n = \frac{4}{a_0^3} \quad (5)$$

Here a_0 is the lattice constant.

7. G_{max} calculation Parameters

Table S3: Parameters for the calculation of G_{max}

Material	Longitudinal Acoustic Velocity (m/s)	Transverse Acoustic Velocity (m/s)	Maximum Longitudinal Phonon Frequency (THz)	Maximum Transverse Phonon Frequency (THz)	Lattice Constant (Å)
Diamond	18000	12820	32	24	3.57
SiC	12500	7200	19	12.5	4.36
Si	9600	5600	12	5	5.43
Ge	5400	3400	7	2.4	5.66
cBN	17000	11800	26	21	3.62
AlN	11300	6300	15	11	4.37
GaN	8080	4150	9	6.5	4.48
TiN	10428	6170	10	8	4.2
HfN	6573	4375	5	4.5	4.525
Ti	6070	3125	7	6	2.95

8. GaN/Diamond HEMT Modelling

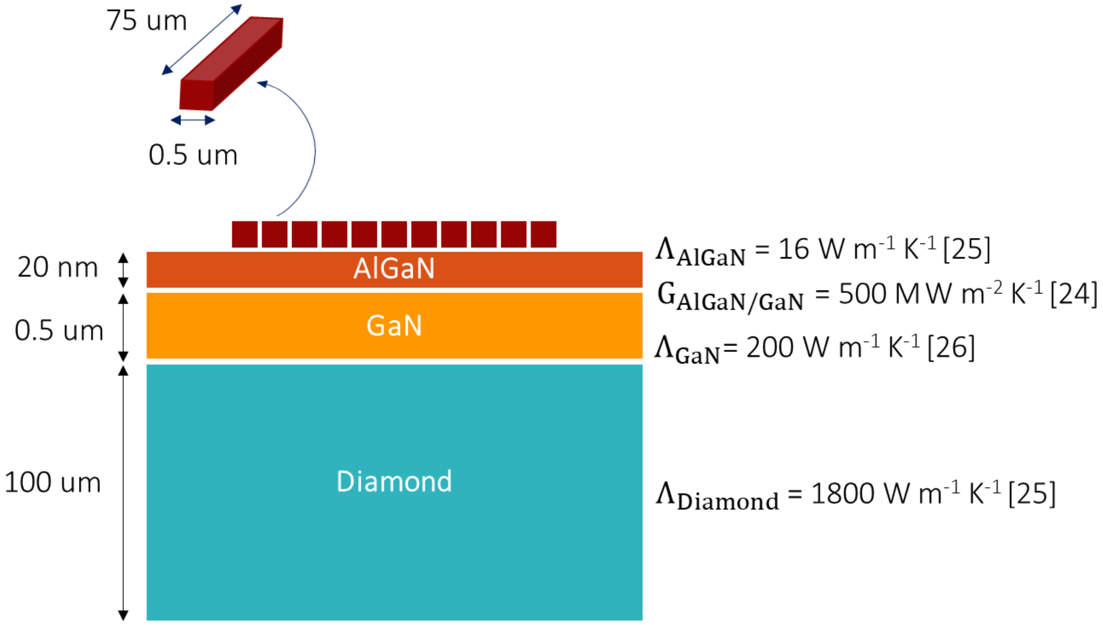

Figure S17. Schematic of the HEMT device geometry we used as an example of how nitride/diamond interface conductance will affect device performance.

Thermal parameters used for modelling the temperature rise in a GaN/Diamond HEMT are detailed in figure S17. We used the interface conductance of AlN/GaN [24] for $G_{AlGaN/GaN}$, since AlGaN is vibrationally similar to AlN, with some of the Al atoms substituted by Ga atoms. The analytical solution from ref [25] includes a 50 um SnAg die-attach layer and a convective heat transfer coefficient of 6.5×10^5 W m^{-2} K^{-1} in the source plane to model the thermal resistance of the die attach and high-performance microchannel cooler.

The analytical solution predicts a resistance R_{sub} = 4.5 m^2 K GW^{-1} for the thermal resistances from the diamond substrate, die-attached layer and convective boundary at the bottom of the die.

The interface conductance between GaN and diamond adds in parallel with the rest of the device's resistance. Therefore, the device temperature rise is given by,

$$\Delta T_{device} = \frac{J}{R_{total}} \tag{6}$$

where J is the power density and R_{total} is the total device thermal resistance,

$$R_{total} = \frac{h_{AlGaN}}{\Lambda_{AlGaN}} + \frac{1}{\mathrm{G}_{\mathrm{AlGaN/GaN}}} + \frac{h_{GaN}}{\Lambda_{GaN}} + \frac{1}{\mathrm{G}_{\mathrm{GaN/Diamond}}} + \mathrm{R}_{\mathrm{sub}} \tag{7}$$

Here, h is the thickness of the layer, Λ is the thermal conductivity of the material and G is the interface conductance between the respective layers.

SUPPLEMENTARY REFERENCES

(1) Hohensee, G. T.; Hsieh, W. P.; Losego, M. D.; Cahill, D. G. Interpreting Picosecond Acoustics in the Case of Low Interface Stiffness. *Review of Scientific Instruments* **2012**, *83* (11). https://doi.org/10.1063/1.4766957.

(2) Costescu, R. M.; Wall, M. A.; Cahill, D. G. Thermal Conductance of Epitaxial Interfaces. *Physical Review B* **2003**, *67* (5), 054302. https://doi.org/10.1103/PhysRevB.67.054302.

(3) Srivastava, A.; Diwan, B. D. Structural and Elastic Properties of ZrN and HfN: Ab Initio Study. *Canadian Journal of Physics* **2014**, *92* (9), 1058–1061. https://doi.org/10.1139/cjp-2013-0377.

(4) Levinshtein, M. E.; Rumyantsev, S.; Shur, M. S. *Properties of Advanced Semiconductor Materials : GaN, AlN, InN, BN, SiC, SiGe*; Levinshteĭn, M. E., Rumyantsev, S. L., Shur, M., Eds.; Wiley: New York, 2001.

(5) Abe, H.; Kato, H.; Baba, T. Specific Heat Capacity Measurement of Single-Crystalline Silicon as New Reference Material. **2011**, *50* (11), 11RG01. https://doi.org/10.1143/jjap.50.11rg01.

(6) Victor, A. C. Heat Capacity of Diamond at High Temperatures. *The Journal of Chemical Physics* **1962**, *36* (7), 1903–1911. https://doi.org/10.1063/1.1701288.

(7) Ushakov, S. v.; Navrotsky, A.; Hong, Q.-J.; van de Walle, A. Carbides and Nitrides of Zirconium and Hafnium. *Materials* **2019**, *12* (17), 2728. https://doi.org/10.3390/ma12172728.

(8) Shomate; Howard, C. Specific Heats at Low Temperatures of TiO, Ti2O3, Ti3O5, and TiN1. *J Am Chem Soc* **1946**, *68* (2), 310–312.

(9) Ditmars, D. A.; Ishihara, S.; Chang, S. S.; Bernstein, G.; West, E. D. Enthalpy and Heat-Capacity Standard Reference Material: Synthetic Sapphire (Alpha-Al2O3) From 10 to 2250 K. *Journal of Research of the National Bureau of Standards* **1982**, *87* (2), 159. https://doi.org/10.6028/jres.087.012.

(10) Victor, A. C.; Douglas, T. B. Thermodynamic Properties of Magnesium Oxide and Beryllium Oxide from 298 to 1,200 ÃƒÆ’Ã¢â‚¬Å¡Ãƒâ€šÃ‚Â°K. *Journal of Research of the National Bureau of Standards Section A: Physics and Chemistry* **1963**, *67A* (4), 325. https://doi.org/10.6028/jres.067A.034.

(11) Arthur, J. S. The Specific Heats of MgO, TiO 2 , and ZrO 2 at High Temperatures. *Journal of Applied Physics* **1950**, *21* (8), 732–733. https://doi.org/10.1063/1.1699748.

(12) Koshchenko, V. I.; Grinberg, Y.; Demidenko, A. F. Thermodynamic Properties of AlN (5-2700 K), GaP (5-1500 K) and BP (5-800 K). *Izv. Akad. Nauk SSSR, Neorg. Mater* **1984**, *20* (11), 1798–1790.

(13) Leonidov, V. Y.; Timofeev, I.; Solozhenko, V.; Rodionov, I. ENTHALPIES OF THE FORMATION OF CUBIC BORON-NITRIDE. *ZHURNAL FIZICHESKOI KHIMII* **1987**, *61* (10), 2851–2852.

(14) Nikanorov, S. ~P.; Kardashev, B. ~K. Elasticity and Dislocation Inelasticity of Crystals. *Moscow Izdatel Nauka* **1985**.

(15) Berman, R.; Hudson, P. R. W.; Martinez, M. Nitrogen in Diamond: Evidence from Thermal Conductivity. *Journal of Physics C: Solid State Physics* **1975**, *8* (21), L430–L434. https://doi.org/10.1088/0022-3719/8/21/003.

(16) Zheng, Q.; Li, C.; Rai, A.; Leach, J. H.; Broido, D. A.; Cahill, D. G. Thermal Conductivity of GaN 71, GaN, and SiC from 150 K to 850 K. *Physical Review Materials* **2019**, *3* (1), 014601. https://doi.org/10.1103/PhysRevMaterials.3.014601.

(17) Kremer, R. K.; Graf, K.; Cardona, M.; Devyatykh, G. G.; Gusev, A. V.; Gibin, A. M.; Inyushkin, A. V.; Taldenkov, A. N.; Pohl, H.-J. Thermal Conductivity of Isotopically Enriched 28Si: Revisited. *Solid State Communications* **2004**, *131* (8), 499–503. https://doi.org/10.1016/j.ssc.2004.06.022.

(18) Maycock, P. D. Thermal Conductivity of Silicon, Germanium, III–V Compounds and III–V Alloys. *Solid-State Electronics* **1967**, *10* (3), 161–168. https://doi.org/10.1016/0038-1101(67)90069-X.

(19) Chen, K.; Song, B.; Ravichandran, N. K.; Zheng, Q.; Chen, X.; Lee, H.; Sun, H.; Li, S.; Udalamatta Gamage, G. A. G.; Tian, F.; Ding, Z.; Song, Q.; Rai, A.; Wu, H.; Koirala, P.; Schmidt, A. J.; Watanabe, K.; Lv, B.; Ren, Z.; Shi, L.; Cahill, D. G.; Taniguchi, T.; Broido, D.; Chen, G. Ultrahigh Thermal Conductivity in Isotope-Enriched Cubic Boron Nitride. *Science (1979)* **2020**, *367* (6477), 555–559. https://doi.org/10.1126/science.aaz6149.

(20) Franco Júnior, A.; Shanafield, D. J. Thermal Conductivity of Polycrystalline Aluminum Nitride (AlN) Ceramics. *Cerâmica* **2004**, *50* (315), 247–253. https://doi.org/10.1590/S0366-69132004000300012.

(21) Kargar, F.; Balandin, A. A. Advances in Brillouin–Mandelstam Light-Scattering Spectroscopy. *Nature Photonics* **2021**, *15* (10), 720–731. https://doi.org/10.1038/s41566-021-00836-5.

(22) Yu, P. Y.; Cardona, M. *Fundamentals of Semiconductors*; Springer Berlin Heidelberg: Berlin, Heidelberg, 2005. https://doi.org/10.1007/b137661.

(23) Stoehr, M.; Seo, H.-S.; Petrov, I.; Greene, J. E. Raman Scattering from Epitaxial HfN Layers Grown on MgO(001). *Journal of Applied Physics* **2006**, *99* (4), 043507. https://doi.org/10.1063/1.2173037.

(24) Wang, Q.; Wang, X.; Liu, X.; Zhang, J. Interfacial Engineering for the Enhancement of Interfacial Thermal Conductance in GaN/AlN Heterostructure. *Journal of Applied Physics* **2021**, *129* (23), 235102. https://doi.org/10.1063/5.0052742.

(25) Bagnall, K. R.; Muzychka, Y. S.; Wang, E. N. Analytical Solution for Temperature Rise in Complex Multilayer Structures With Discrete Heat Sources. *IEEE Transactions on Components, Packaging and Manufacturing Technology* **2014**, *4* (5), 817–830. https://doi.org/10.1109/TCPMT.2014.2299766.

(26) Cheng, Z.; Mu, F.; Yates, L.; Suga, T.; Graham, S. Interfacial Thermal Conductance across Room-Temperature-Bonded GaN/Diamond Interfaces for GaN-on-Diamond Devices. *ACS Applied Materials and Interfaces* **2020**, *12* (7), 8376–8384. https://doi.org/10.1021/acsami.9b16959.

(27) Archilla, J. F. R.; Coelho, S. M. M.; Danie Auret, F.; Nyamhere, C.; Dubinko, V. I.; Hizhnyakov, V. Experimental Observation of Intrinsic Localized Modes in Germanium. *Springer Series in Materials Science* **2015**, *221*, 343–362. https://doi.org/10.1007/978-3-319-21045-2_14.

(28) Strauch, D. AlN: Phonon Dispersion Curves, Phonon Density of States, Phonon Frequencies, Phonon Eigenvectors; 2011; pp 84–93. https://doi.org/10.1007/978-3-642-14148-5_60.

(29) Pavone, P.; Karch, K.; Schiitt, O.; Windl, W.; Strauch, D.; Giannozzi, P.; Baroni, S. *Ab. Initio Lattice Dynamics of Diamond*; Vol. 48.

(30) Nipko, J. C.; Loong, C. K.; Balkas, C. M.; Davis, R. F. Phonon Density of States of Bulk Gallium Nitride. *Applied Physics Letters* **1998**, *73* (1), 34–36. https://doi.org/10.1063/1.121714.

(31) Parlinskí Parlinskí, * K. *Lattice Dynamics of Cubic BN*; 2001; Vol. 328.
